# Essential Oils from Neotropical *Piper* Species and Their Biological Activities

**DOI:** 10.3390/ijms18122571

**Published:** 2017-12-14

**Authors:** Joyce Kelly da Silva, Rafaela da Trindade, Nayara Sabrina Alves, Pablo Luís Figueiredo, José Guilherme S. Maia, William N. Setzer

**Affiliations:** 1 Programa de Pós-Graduação em Biotecnologia, Universidade Federal do Pará, Belém 66075-900, Brazil; rcabral@ufpa.br (R.d.T.); nayra.sab02@gmail.com (N.S.A.); 2 Programa de Pós-Graduação em Química, Universidade Federal do Pará, Belém 66075-900, Brazil; pablolbf@ufpa.br (P.L.F.); gmaia@ufpa.br (J.G.S.M.); 3 Department of Chemistry, University of Alabama in Huntsville, Huntsville, AL 35899, USA; wsetzer@chemistry.uah.edu; 4 Aromatic Plant Research Center, 615 St. George Square Court, Suite 300, Winston-Salem, NC 27103, USA

**Keywords:** piperaceae, neotropics, antimicrobial, cytotoxic, antiprotozoal, anticholinesterase, anti-inflammatory, analgesic

## Abstract

The *Piper* genus is the most representative of the Piperaceae reaching around 2000 species distributed in the pantropical region. In the Neotropics, its species are represented by herbs, shrubs, and lianas, which are used in traditional medicine to prepare teas and infusions. Its essential oils (EOs) present high yield and are chemically constituted by complex mixtures or the predominance of main volatile constituents. The chemical composition of *Piper* EOs displays interspecific or intraspecific variations, according to the site of collection or seasonality. The main volatile compounds identified in *Piper* EOs are monoterpenes hydrocarbons, oxygenated monoterpenoids, sesquiterpene hydrocarbons, oxygenated sesquiterpenoids and large amounts of phenylpropanoids. In this review, we are reporting the biological potential of *Piper* EOs from the Neotropical region. There are many reports of *Piper* EOs as antimicrobial agents (fungi and bacteria), antiprotozoal (*Leishmania* spp., *Plasmodium* spp., and *Trypanosoma* spp.), acetylcholinesterase inhibitor, antinociceptive, anti-inflammatory and cytotoxic activity against different tumor cells lines (breast, leukemia, melanoma, gastric, among others). These studies can contribute to the rational and economic exploration of *Piper* species, once they have been identified as potent natural and alternative sources to treat human diseases.

## 1. Introduction

The genus *Piper* L. has approximately 2000 species distributed in the pantropical region, in the Neotropics occurring from northern Mexico to Chile and Argentina. The Andean slopes, Central American lowlands and Central Amazonia have been considered as centers of high species richness for the genus [1,2]. *Piper* belongs to Piperaceae, classified in the order Piperales, Magnoliids clade included in angiosperm basal group [3]. Phylogenetic studies have confirmed the monophyly of the group with eight subgenera recognized in the Neotropics: *Enckea*, *Macrostachys*, *Ottonia*, *Peltobryon*, *Piper*, *Pothomorphe*, *Radula* and *Schilleria* [4].

Plants of the genus *Piper* are easy recognized in the field by their nodose shoots, inflorescences spikes, and the typical “spicy” or aromatic smell [4]. They can be herbs, shrubs and less often lianas of annual or perennial habits; aromatics; glabrous or with varied indumentum, frequently gland-dotted, with nodose stems. Leaves are mostly alternate, sometimes opposite, simple, sessile or petiolate, with variable size, shape and venation. Inflorescences are terminal, leaf opposed or axillary, commonly spike solitary, umbellate or paniculate, erect, pendent or recurved, variable in size. Flowers are very small and numerous, generally monoic, perianthless, variable in shape; stamens usually 2–6, arising near the base of the ovary with filaments free and generally short, anthers with 1, 2, or 4 thecae, laterally or apically dehiscent, deciduous after pollination; gynoecium with superior ovary, with 1, 3, or 5 fused carpels, 1-locular. Fruit is a small berry or drupe, variously shaped, with a thin pericarp and sometimes hardened endocarp; seed small, solitary [5].

Since prehistoric times *Piper* spp. have been used by man, manly as spices, in mystical and cultural activities and in folk medicine to treat many diseases. For example, a decoction of the leaves of *P. cavalcantei* is considered by native Amazon people as excellent antipyretic and analgesic [6]; *P. marginatum*, which is used by indigenous communities in Central America, the Antilles, and South America for gastrointestinal problems [7]; and *P. umbellatum* that is traditionally used as an anti-inflammatory in Brazil, to treat wounds in Cuba, and to treat fever in Peru [8]. In the literature, there have been many studies about Asian *Piper* species, but a large proportion of the information has been generated from Latin American species, pointing to an enormous diversity of chemical compounds associated with its diversity of biological activities [9,10]. In tropical countries, many species of *Piper* are used by traditional societies by their anti-inflammatory and analgesic properties, and have large potential for the pharmaceutical industry [11].

*Piper* species produce a number of metabolic classes with diverse biological activities [10]. The essential oils extracted from different organs of many specimens is constituted mainly of monoterpene hydrocarbons (e.g., α-pinene, myrcene, limonene, α-terpinene, *p*-cymene), oxygenated monoterpenoids (e.g., 1,8-cineole, linalool, terpinen-4-ol, borneol, camphor), sesquiterpene hydrocarbons (e.g., β-caryophyllene, α-humulene, germacrene D, bicyclogermacrene, α-cubebene), oxygenated sesquiterpenoids (e.g., spathulenol, (*E*)-nerolidol, caryophyllene oxide, α-cadinol, *epi*-α-bisabolol) and phenylpropanoids (e.g., safrole, dillapiole, myristicin, elemicin, (*Z*)-asarone, eugenol) (see Appendix A and Appendix B) [12,13]. The literature reports that some tropical species of *Piper* have presented high yields of essential oils [14]. The yield of essential oil and its major volatile components in *Piper* spp. may vary according to geographical region and environmental factors, being conditioned mainly to its different chemotypes [14,15,16]. *P. xylosteoides* leaves presented a yield of 1.8% with the main compound being myrcene (31%), which is largely used in the food and cosmetic industry [17]. *P. divaricatum* had yielded around 46.0%, eugenol, a compound that has been used as an anesthetic for the sedation of fish, in addition to being widely used as a local anesthetic during endodontic and restorative treatments by dentists [18,19]. Essential oils of high yield, along with the presence of volatile compounds of economic value, are valued by the international market due to their wide importance to the pharmacological, cosmetics, and cleaning products industries, among other applications [14]. *Piper* has been a model genus for ecological and evolutionary studies, and *Piper* species are considered important due their association with frugivorous bats [4]. A suite of insect herbivores feeds on the leaves of *Piper*, the ripening fruits are attacked by a variety of seed predators, and ripe fruits provide food for frugivorous bats and birds, and other animals surely use *Piper* fruits as food at least occasionally [11].

Taxonomic difficulties in the genus *Piper* are related to the great variability and minute nature of their flowers [20]. Due to the pronounced pharmacological value and worldwide demand of *Piper* species, it is imperative to make efforts to the secure botanical identification, to conserve the germplasm, and to allow genetic improvement [21]. The use of biochemical and genetic markers as well as chemical studies of specimens have been shown to be effective methods [22,23,24]. In this review, we report the biological activities and the chemical compositions of *Piper* species native to the Neotropics.

## 2. Volatile Profiles

The essential oil compositions (major components) of Neotropical *Piper* species are summarized in Appendix A. For *Piper* species where several different essential oils were collected, there seems to be wide variation in the compositions. Thus, for example, *P. aduncum* leaf oils can be rich in monoterpenoids such as 1,8-cineole [25], sesquiterpenoids such as (*E*)-nerolidol [26], or dominated by phenylpropanoids like dillapiole [27,28,29,30] or asaricin [31]. At least nine different chemotypes of *P. aduncum* have been characterized [32]. Likewise, *P. amalago* has shown wide variation in essential oil composition with monoterpenoid-rich [31,33] and sesquiterpenoid-rich [34,35] chemotypes. Dihydroagarofurans have dominated several leaf oils of *P. cernuum* [31,36,37] while other samples have shown monoterpene and sesquiterpene hydrocarbons as major components [35,38,39]. *Piper divaricatum* has shown a eugenol/methyleugenol chemotype [40,41,42,43] as well as a safrole chemotype [44]. Phenylpropanoids have characterized the leaf essential oils of many samples of *P. marginatum* from Brazil [41,45], but even these show wide variation in the phenylpropanoid concentrations. Chemical structures for the major *Piper* essential oil components are shown in Appendix B.

Geographical location and habitat likely affects the chemical compositions. For example, the leaf essential oil compositions of *P. hispidum* from Cuba [46] was rich in eudesmols, while a sample from Panama was dominated by dillapiole [47], and a sample from Colombia had (*E*)-nerolidol as the major component [48]. *Piper umbellatum* essential oils from Monteverde, Costa Rica [49], and from São Paulo, Brazil [31], were rich in sesquiterpene hydrocarbons, while the essential oil from the Escambray Mountains of Cuba was rich in camphor and safrole [50]. The wide variations in essential oil compositions certainly impacts the biological activities of the *Piper* oils (see Appendix A) and likely affects the traditional medicinal uses of the plants in their native habitats.

## 3. Biological Activities

### 3.1. Antibacterial and Antifungal Activity

The need to combat microbial resistance to present day antibiotics has boosted efforts for bioprospecting to identify new antibacterial and antifungal agents [51]. The potential of *Piper* essential oils against pathogenic Gram-positive bacteria, such as *Staphylococcus aureus*, *Bacillus cereus*, *Bacillus subtilis* and *Streptococcus pyogenes*, as well as Gram-negative microorganisms, which include *Escherichia coli*, *Klebsiella pneumoniae*, *Pseudomonas aeruginosa* and *Acinetobacter baumanii*, has been reported in several published investigations. Essential oils (EOs) from leaves of *P. abutilodes*, *P. aduncum*, *P. marginatum*, and *P. molicomum* were tested against *Escherichia coli* (EPEC 0031-2) and had minimal inhibitory concentrations (MICs) ranging from 500 µg/mL to 1000 µg/mL [52]. *P. regnellii* oils from leaves had MIC of 300 µg/mL against the serotype EPEC 0031-2. Antimicrobial assays were performed by microdilution method, but EO compositions were not reported [52]. EOs from leaves of *P. aduncum* var. *ossanum* (Bauta, Artemisa Province, Cuba) were mainly composed of piperitone (20.1%), viridiflorol (13.0) and camphor (13.9%) and had high activity against *Staphylococcus aureus* with an IC_50_ value of 39.5 µg/mL [53].

The antimicrobial activity was evaluated against *Bacillus cereus* and *S. aureus* using broth dilution assay for different *Piper* species collected in Monteverde (Costa Rica) [34]. The oils from leaves of *Piper* sp. aff. *aereum*, *P. bredemeyeri*, *P. oblanceolatum* displayed a MIC value of 78 μg/mL against *Bacillus cereus*, while *P. fimbriulatum* was more active (MIC, 39 μg/mL). In addition, *Piper* sp. aff. *aereum* showed activity against *S. aureus* (MIC, 78 μg/mL). The main compounds in these oils were mostly sesquiterpenoids and monoterpenoids: *Piper* sp. aff. *aereum* oil was composed of guaiol (41.2%), α-cadinol (9.2%) and δ-cadinene (7.3%). *P. bredemeyeri* showed β-elemene (34.0%), β-caryophyllene (24.2%) and germacrene D (21.7%) as major compounds. *P. fimbriulatum* showed germacrene D (32.9%), α-pinene (10.2%) and δ-elemene (9.4%), while *P. oblanceolatum* was rich in linalool (11.3%), δ-amorphene (9.0%) and germacrene D (8.9%) [34].

*Piper caldense* oils from different tissues displayed as major components α-cadinol (19.0%), α-muurolol (9.0%), and thujopsan-2β-ol (7.4%) (leaves); terpinen-4-ol (18.5%), α-terpineol (15.3%), and α-cadinol-2β-ol (9.8%) (stems); and pentadecane (35.7%), valencene (10.5%), and selina-3,7(11)-diene (5.4%) (roots). The samples were tested against *E. coli*, *Klebsiella pneumoniae*, *Pseudomonas aeruginosa*, *S. aureus* and *B. subtilis* using agar diffusion assay and showed moderate to weak bacterial activity with MIC value of 325 or 750 μg/mL in comparison to gentamicin (5.0 μg/mL) [54]. *P. cernuum* and *P. regnellii* EOs from leaves inhibited growth of *S. aureus* and *Candida albicans* when evaluated by agar diffusion assay. The main compounds in *P. cernuum* oil were bicyclogermacrene (21.9%), β-caryophyllene (20.7%) and α-pinene, while *P. regnelli* essential oil was dominated by myrcene (52.6%), linalool (15.9%) and β-caryophyllene (8.5%) [38].

The chemical composition by CG-MS and antimicrobial activity using agar dilution technique of *P. cernuum* oils were evaluated seasonally [37]. The main compounds identified were *trans*-dihydroagarofuran (30 to 36.7%), 4-*epi*-*cis*-dihydroagarofuran (11.2 to 13.4%), and γ-eudesmol (7.64 to 11.6%). The EOs showed significant activity against *S. aureus* (MIC 780 μg/mL, and for spring MIC, 1560 μg/mL), *B. subtilis* (MIC 780 μg/mL) and *Streptococcus pyogenes* (MIC 48–390 μg/mL) [37]. *P. diospyrifolium* EO from leaves, composed of *cis*-eudesma-6,11-diene (21.1%), β-caryophyllene (16.8%), and γ-muurolene (10.6%), was tested using agar diffusion assay and it showed significant potential against clinical fungal strains of *C. albicans*, *C. parapsilosis*, *C. tropicalis* in comparison to nystatin [55]. *P. malacophyllum* oil, composed mainly of camphor (32.8%), camphene (20.8%), and (*E*)-nerolidol (9.1%), displayed a weak activity against *S. aureus*, *P. aeruginosa*, *Acinetobacter baumanii* (MIC 3700 µg/mL), *B. cereus*, and *E. coli* (MIC 1850 µg/mL) using the broth microdilution method [56]. Furthermore, *P. ilheusense* oil from leaves was made up of β-caryophyllene (11.8%), patchouli alcohol (11.1%), and gleenol (7.5%), and was active against the fungi *C. albicans*, *C. parapsilosis* and *C. krusei,* and it partially combated *B. subtilis* and *S. aureus* [57]. *P. tuberculatum* EO from seeds was mostly composed of β-elemene, β-caryophyllene and β-farnesene, among others; microdilution assays indicated that this oil inhibited growth of *S. aureus* and *B. subtilis*. However, neither the EO composition nor MIC values were reported [58].

Several *Piper* oils have been reported to be effective to combat fungal species of *Cryptococcus*. *P. aduncum* oil from leaves was composed of linalool (31.8%), bicyclogermacrene (11.3%), and (*E*)-nerolidol (10.3%), and showed activity against *Cryptococcus neoformans* (MIC 62.5 μg/mL) [35]. Similarity, *P. gaudichaudianum* oil, rich in α-humulene (23.4%), β-caryophyllene (15.6%), and viridiflorene (8.1%), showed significant antifungal activity against *C. krusei* (MIC 31.25 μg/mL). EO from leaves of *P. solmsianum* was mostly composed of (*E*)-isoelemecin (53.5%), spathulenol (5.2%), and *epi*-α-muurolol (4.6%), and had significant activity against *C. neoformans* (MIC 62.5 μg/mL). These analyses were performed by means of broth dilution assays [35].

The activity of *Piper* EOs has been evaluated by direct bioautography on TLC plates against *Cladosporium cladosporioides* and *C. sphaerospermum* [59,60,61,62]. *Cladosporium* spp. are strong airborne contaminants that cause allergies and other serious diseases of the respiratory tract [63,64]. The oils from fruits of *P. aduncum* and *P. tuberculatum* had a higher activity in comparison to miconazole and nystatin against *Cladosporium cladosporioides* and *C. sphaerospermum*, respectively. Both oils displayed a detection limit (DL) of 10 μg [59]. Oils of *P. aleyreanum*, *P. anonifolium*, *P. hispidum* and *P. divaricatum*, collected from the Brazilian Amazon, showed a higher activity with DL values between 0.1 and 5.0 μg [61]. These oils were mainly composed of terpenoids as β-elemene (16.3%), bicyclogermacrene (9.2%), and δ-elemene (8.2%) (*P. aleyreanum*); selin-11-en-4α-ol (20.0%), β-selinene (12.7%), and α-selinene (11.9%) (*P. anonifolium*); and δ-3-carene (9.1%), β-caryophyllene (10.5%), and α-humulene (9.5%) (*P. hispidum*). *P. divaricatum* oil, on the other hand, was dominated by phenylpropanoids methyleugenol (63.8%) and eugenol (23.6%) [40,61]. Essential oils of *P. cernuum* (fruits), *P. solmsianum* (leaves), and *P. crassinervium* (leaves) displayed moderated and weak activity against these pathogens. Germacrene D (14.0%) and spathulenol (9.8%) were the main components of *P. cernuum* and *crassinervium* oils, while *P. solmsianum* was dominated by (*E*)-isoelemicin (53.9%) [60].

*Piper aduncum* oil, chemotype dillapiole (85.9%), and isolated dillapiole exhibited antifungal activity against pathogenic skin microorganisms [30]. The samples were assayed by the microdilution method and displayed MIC values of 500 μg/mL for the strains of *Trichophyton mentagrophytes* (ATCC 9533 and clinical isolate), *T. rubrum*, and *Epidermophyton floccosum*. For clinical isolates of *Microsporum canis*, *M. gypseum*, and *Aspergillus fumigatus* (ATCC 40152 and clinical isolate), the MIC values were 250, 250 and 3.9 μg/mL, respectively. Minimum fungicidal concentration (MFC) values displayed a range of 15.6 to 1500 μg/mL for all samples. The EO and its dillapiole-rich fraction demonstrated significant antifungal activity against dermatophytes, filamentous fungi, and potent antifungal activity against non-dermatophyte filamentous fungi [30]. In addition, *P. aduncum* oils from the aerial parts of chemotype dillapiole (45.9%), (*E*)-β-ocimene (19.0%), and piperitone (8.4%), also inhibited *Trichophyton* species at 500 µg/mL [65]. *P. cernuum* oil, rich in *trans*-dihydroagarofuran (30.0–36.7%), 4-*epi*-*cis*-dihydroagarofuran (11.2–13.4%), and γ-eudesmol (8.3–13.3%), was active against *Microsporum gypseum* (MIC, 48–390 μg/mL), *T. mentagrophytes* (MIC, 48–195 μg/mL), *T. rubrum* (MIC, 48–195 μg/mL), *E. flocosum* (MIC 48–195 μg/mL) and opportunist yeast *Cryptococcus neoformans* (MIC 48 μg/mL) [37]. *P. malacophyllum* oil was composed of camphor (32.8%), camphene (20.8%), and (*E*)-nerolidol (9.1%), and it showed moderate activity compared to ketoconazole against *T. mentagrophytes* and *C. neoformans*, the causal agent of meningoencephalitis in immunocompromized patients [56].

### 3.2. Antiprotozoal Activity

Parasitic protozoal diseases are the major economic and public health problems in the world causing high rates of human morbidity and mortality in developing countries [32]. The prevalence of these diseases is higher in the tropics, where a significant number of deaths are attributed to leishmaniasis, malaria, and trypanosomiasis [66]. *Piper* species have been reported as good sources of antiparasitic compounds [67].

Studies carried out with the essential oils of *Piper* species showed that *P. aduncum* leaf EO, containing (*E*)-nerolidol (25.2%) and linalool (13.42%), had an inhibitory effect after 24 h on the growth of *Leishmania braziliensis* promastigotes (IC_50_, 77.9 μg/mL) and (*E*)-nerolidol presented a similar inhibitory effect (IC_50_, 74.3 μg/mL) [67,68]. *P. aduncum* leaf EO from two localities in Cuba (Bauta and Ceiba) were active against *L. amazonensis* (IC_50_, 19.3 and >64 μg/mL, respectively), in both EOs, the major components were piperitone, viridiflorol, and camphor [53]. *P. angustifolium* leaf EO, dominated by spathulenol (23.8%) and caryophyllene oxide (13.1%), was effective against intra-cellular amastigotes of *L. infantum*, the etiological agent of visceral leishmaniasis (IC_50_, 1.4 μg/mL) [69]. *P. aduncum* and *P. diospyrifolium* leaf EOs displayed high activity against of axenic amastigote forms of *L. amazonensis* (IC_50_, 76.1 μg/mL and 36.2 μg/mL, respectively) and were more selective for the parasite than for the mammalian macrophages [70]. The main constituents of *P. aduncum* EO were bicyclogermacrene (20.9%), (*E*)-β-ocimene (13.9%), and (*Z*)-β-ocimene (7.0%), while *P. diospyrifolium* oil was rich in selin-11-en-4α-ol (17.7%), β-caryophyllene (7.4%), and γ-gurjunene (6.9%).

The EO from fresh leaves and inflorescences of *P. claussenianum* showed high activity against a strain of *L. amazonensis*, the leaf EO, rich in (*E*)-nerolidol (81.4%), had greater inhibition on the growth of *L. amazonensis* than the inflorescences EO, which was rich in linalool (50.2%) (IC_50_ 30.4 μg/mL and 1328 μg/mL, respectively) [71]. *P. demeraranum* and *P. duckei* oils inhibited the growth of promastigote forms of two species of Leishmania (IC_50_, 15.2 and 22.7 μg/mL, respectively) with greater activity against *L. guyanensis* than *L. amazonenis* [72]. The main constituents of *P. demeraranum* oil were β-elemene (33.1%), limonene (19.3%), and bicyclogermacrene (8.8%), and *P. duckei* β-caryophyllene (27.1%), γ-eudesmol (17.9%), and germacrene D (14.7%).

The *P. aduncum* EOs, chemotype piperitone (19.0–23.7%), camphor (9.4–17.1%) and viridiflorol (13.0–14.5%), obtained from the aerial parts, showed an inhibitory effect on the growth of Plasmodium falciparum with IC_50_ value ranging from 1.3 to 2.8 μg/mL [32,53]. The essential oils from *P. claussenianum* inflorescences and *P. lucaeanum* leaves, and the pure isolated (*E*)-nerolidol obtained from inflorescenses of *P. claussenianum*, showed a 70% decrease in the growth of chloroquine-resistent (W2) *P. falciparum*, when tested at a concentration of 25 mg/mL [73]. The *P. claussenianum* EO was dominated by linalool (56.5%) followed by (*E*)-nerolidol (23.7%) and α-humulene (2.4%), and *P. lucaeanum* was rich in α-pinene (30.0%), α-zingiberene (30.4%) and β-sesquiphellandrene (11.1%).

The anti-trypanosomal activities have been reported for different chemotypes of *P. aduncum* EOs. The effect of *P. aduncum* EO rich in (*E*)-nerolidol (25.2%), linalool (13.4%) and spathulenol (l5.3%), was analyzed against different developmental forms of Trypanosoma cruzi. The oil was active after 24 h against cell-derived (IC_50_, 2.8 μg/mL), metacyclic trypomastigotes (IC_50_ 12.1 μg/mL), and intracellular amastigotes (IC_50_, 9.0 μg/mL) [68]. The chemotype piperitone (23.7%), camphor (17.1%), and viridiflorol (14.5%) also exhibited activity against *T. cruzi* (IC_50_ 2.0 μg/mL) [32]. In addition, *P. aduncum* leaf EOs from two localities in Cuba were active against *T. brucei* and *T. cruzi* with IC_50_ value of approximately 8.0 μg/mL [53]. The major components were piperitone (20.1–19.0%), viridiflorol (13–18.8%) and camphor (13.9–9.4%) in the specimens.

In addition to inhibition of the parasites themselves, inhibition of key protozoal protein targets by essential oils has been investigated [74]. *P. bredemeyeri* leaf essential oil inhibited cruzain, the cysteine protease from Trypanosoma cruzi, with IC_50_ of 0.96 μg/mL [34]. *P. bredemeyeri* EO was composed largely of the sesquiterpene hydrocarbons β-elemene (34.0%), β-caryophyllene (24.2%), germacrene D (21.7%), and germacrene A (13.2%). β-Caryophyllene and germacrene D have both shown cruzain inhibitory activity with IC_50_ values of 32.5 and 22.1 μg/mL, respectively, and a 1:1 binary mixture of these two compounds showed synergistic inhibitory activity (IC_50_ = 9.91 μg/mL) [75].

### 3.3. Anticholinesterase Potential

Acetylcholinesterase (AChE) is an enzyme involved in the termination of impulse transmission by quick hydrolysis of the neurotransmitter acetylcholine (ACh). The AChE potential of drugs is inhibition of this enzyme from breaking down ACh, increasing the level and duration of the neurotransmitter activity [76]. For this reason, studies aiming to discover compounds with anticholinesterase potential are relevant. However, there have been few investigations with this focus in Neotropical regions. The EOs from aerial parts of *Piper* species from the Brazilian Amazon displayed a high activity when evaluated by bioautographic method. All samples had a detection limit (DL) value of 0.01 ng, about one hundred times more effective than the standard physostigmine (DL = 1.0 ng). *P. hispidum* and *P. anonifolium* oils were mainly composed of sesquiterpenoids, such as selin-11-en-4α-ol, β-selinene, α-selinene, β-caryophyllene, and α-humulene [61]. In contrast, EOs from the aerial parts of *P. callosum* and *P. marginatum* were mainly composed of phenylpropanoids, such as safrole and 3,4-methylenedioxypropiophenone (propiopiperone) [62]. Although there are limited data on AChE activity of *Piper* essential oils from the Neotropics, a significant amount of research has been performed on Old World *Piper* essential oils [77,78,79,80,81].

### 3.4. Anti-Inflammatory and Antinociceptive Effects

Although a considerable number of analgesic and anti-inflammatory drugs are available for the treatment of pain and inflammation, there is a continuous search for new compounds, due to the fact that some current drugs lead to adverse reactions and have low efficacy [82]. Plants used in folk medicine, including essential oils, have been shown to be promising new sources of anti-inflammatory and antinociceptive drugs [83,84,85,86,87].

*Piper glabratum* leaf EO indicated β-pinene (13.0%), longiborneol (12.0%), and α-pinene (9.7%) as the main compounds. Anti-inflammatory activity was detected by inhibition of leukocyte migration (100, 300, 700 mg/kg) and the protein extravasation into the pleural exudates (700 mg/kg) with no clinical signs of toxicity [88]. *P. vicosanum* EO minimized edema formation and inhibited leukocyte migration using the carrageenan-induced edema and pleurisy models at doses of 100 and 300 mg/kg [89]. The oil displayed a pronounced anti-inflammatory potential, with no acute toxicity or genotoxicity; its main compounds were γ-elemene (14.2%), α-alaskene (13.4%) and limonene (9.1%).

The *P. aleyreanum* EO was tested for antinociceptive activity on two phases of pain model, early neurogenic and the second inflammatory, by formalin-induced pain through the administration of 20 mL of 2.5% formalin solution by intraplanar injection in mice [90]. The effect was significantly more pronounced on the second phase. The ID_50_ values for each phase were 281.2 and 70.5 mg/kg and the inhibitions observed were 75% and 99% at a dose of 1000 mg/kg, for the first and second phases, respectively. The main compounds of *P. aleyreanum* oil were caryophyllene oxide (11.5%), β-pinene (9.0%), and spathulenol (6.7%) [90]. *P. mollicomum* and *P. rivinoides* EOs were evaluated for their antinociceptive activity using the acetic acid-induced writhing in mice [91]. At a dose of 1 mg/kg, the samples inhibited 50.2% and 20.9% of the writhing in mice, respectively. The main constituents of *P. mollicomum* were (*E*)-β-ocimene (14.0%), germacrene B (13.3%), and (*Z*)-β-ocimene (12.1%), and for *P. rivinoides* were α-pinene (32.9%), β-pinene (24.7%), and β-caryophyllene (7.6%). Oral administration of both oils did not induce any apparent acute toxicity [91].

### 3.5. Cytotoxic Activity

EOs with anticancer potential can act by two ways: chemoprevention and cancer suppression. Hence, EOs causing apoptosis in tumor cells are valuable resources in cancer suppression [92,93,94]. Essential oils from *Piper* species have been reported to possess antineoplastic properties against different cancer cells lines such as human colorectal carcinoma, breast tumor, melanoma, gastric tumor, leukemia, among others. The EO of *P. aequale*, rich in δ-elemene (19.0%), β-pinene (15.6%) and α-pinene (12.6%), showed significant cytotoxic activity against human colorectal carcinoma (HCT-116, IC_50_ 8.69 µg/mL) and human gastric tumor (ACP 03, IC_50_ 1.54 µg/mL) cell lines [95]. After 72 h of treatment, the oil has induced apoptosis in the gastric tumor cells in all tested concentrations (0.75–3.0 μg/mL). The EOs of *P. biasperatum*, *P. glabrescens*, *P. imperiale*, *P. oblanceolatum* and *Piper* sp. aff. *aereum* showed greater than 90% mortality against human breast adenocarcinoma cells (MCF-7) at a concentration of 100 µg/mL [34]. The main compounds identified in these samples were β-elemene (46.6%), limonene (56.6%), β-caryophyllene (25.5%) and linalool (11.3%), respectively.

*Piper aleyreanum* oil, rich in β-elemene (16.3%), bicyclogermacrene (9.2%) and δ-elemene (8.2%), showed strong cytotoxic activity (IC_50_ 7.4 μg/mL) against human melanoma (SkMEL 19) [61]. The oil from leaves and branches of *P. cernuum* displayed a broad cytotoxicity spectrum (IC_50_ < 30 μg/mL) including murine melanoma (B16F10-Nex2), human melanoma (A2058), human glioblastoma (U87-MG), human cervical tumor (HeLa), and human myloid leukemia (HL-60) cells [39,96]. These oils showed large amounts of β-elemene (30.0%), bicyclogermacrene (19.9%) and β-caryophyllene (16.3%) in leaves and camphene (46.4%), α-terpineol (11.6%) and carvacrol (11.6%) in the branches.

*Piper hispidum* oil, rich in α-pinene (15.3%) and β-pinene (14.8%), induced the death of cancer cell lines such as human cervical (HeLa), human lung (A-549), human breast (MCF-7) with average IC_50_ values of 36 μg/mL [97]. The EO from *P. regnellii* leaves displayed an expressive cytotoxic activity against human cervical cells carcinoma (HeLa) with IC_50_ value of 13 μg/mL [98]. In addition, the activity was determined to be due to its main compounds germacrene D (51.4%), β-caryophyllene (9.5%) and α-chamigrene (11.3%), which demonstrated IC_50_ values of 11.0, 7.0 and 32.0 μg/mL, respectively.

## 4. Composition-Bioactivity Correlation

A multivariate statistical analysis was carried out in order to discern any relationship between chemical profiles and biological activities for *Piper* essential oils (described in Appendix A). The total percentage of compound classes (monoterpene hydrocarbons (MH), oxygenated monoterpenoids (OM), sesquiterpene hydrocarbons (SH), oxygenated sesquiterpenoids (OS) and phenylpropanoids (PP) to each oil was extracted from original citation (Table A1). These data were used as variables (see Appendix C). The values were normalized and submitted to Principal Component Analysis (PCA) using the Minitab software (free 390 version, Minitab Inc., State College, PA, USA).

**Figure A5:**
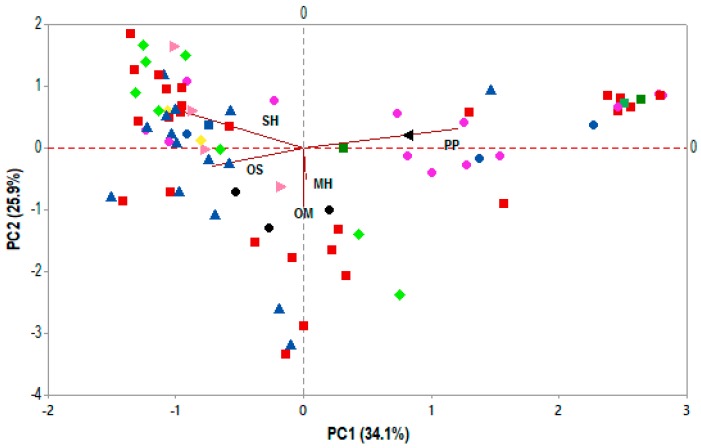
■: Antimicrobial (fungal and bactericidal), ♦: Cytotoxic activity, ▲: Antiprotozoal (*Trypanosoma spp.* and *Leishmania spp.*), ●: Inseticidal (*Aedes aegypti*), ■: Acaricidal, ♦: Anti-inflammatory; ●: Anticholinesterase, ●: Antinociceptive, ■: Central nervous system depressant, ►: Antimicrobial and cytotoxic, ◄: Fungicidal and Anticholinesterase.

The antimicrobial activity (fungicidal and bactericidal) displayed a correlation to all compound classes identified in *Piper* species. However, the cytotoxic activity is related to higher amounts of sesquiterpene hydrocarbons (0–94.9%), monoterpene hydrocarbons (0–83.7%). The antiprotozoal activity is related to *Piper* oils with low concentrations of monoterpene hydrocarbons (<29.9%) and high concentrations of oxygenated monoterpenoids (0–50.3%), sesquiterpene hydrocarbons (3.3–76.0%) and oxygenated sesquiterpenoids (0–86.2%). For this activity, only the *P. auritum* oil, which was rich in phenylpropanoids (88.5%), showed activity against *Leishmania* spp. *Piper* oils described as rich in phenylpropanoids and sesquiterpenes hydrocarbons displayed high insecticidal and acaricidal activities. In addition, the amounts of phenylpropanoids and sesquiterpenoids (hydrocarbons and oxygenated) are related to acetylcholinesterase inhibition. The anti-inflammatory effects were mostly observed in *Piper* oils rich in sesquiterpene hydrocarbons (16.2–62.6%) while antinociceptive effects cover oils that showed monoterpene hydrocarbons (16.6–65.0%) as main compounds. The essential oil composition and biological activity correlations are summarized in Figure A5 and Table 1.

## 5. Conclusions

The *Piper* genus has shown great biodiversity in the Neotropics, and essential oils from *Piper* species have likewise demonstrated abundant chemical diversity. The chemical diversity of *Piper* essential oils has led to a myriad of traditional medicinal uses as well as numerous biological activities. The promise of *Piper* essential oils to treat human diseases, infections, and suffering has already been realized, and the future exploration of this genus shows much promise). The expectation that *Piper*'s essential oils can be used to treat diseases, infections and human suffering is already a reality, and the future economical exploration of some species of this genus seems to us as very promising.

## Figures and Tables

**Table 1:** Relationship between biological activity and compound classes presents in the *Piper* oils obtained by PCA analysis.

Activity	Classes (%)				
	MH	OM	SH	OS	PP
Antimicrobial	0–70.2	0–51.4	0–99.8	0–86.2	0–98.0
Cytotoxic	0–83.7	0–23.2	0–94.9	0–29.5	0–6.7
Antiprotozoal	0–29.9	0–50.3	3.3–76.0	0–86.2	0–88.5
Insecticidal	0–44.3	0–12.6	0–66.0	0–45.4	0–98.8
Enzymatic	6.9–18.5	1–4.2	4.9–52.2	2.1–17.5	0–80.8
Anti-inflammatory	16.4–25.8	0–16.4	16.2–62.6	20.8–28.3	0–0.2
Antinociceptive	16.6–65.9	0.8–16.4	16.2–33.2	4.8–28.3	0–0.6

**Figure A1:**
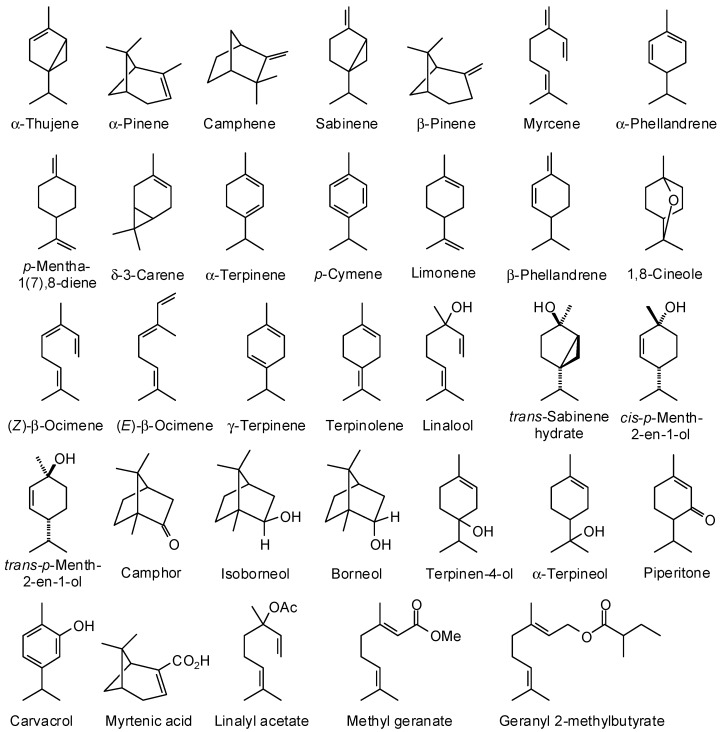
Major monoterpenoids found in Neotropical *Piper* species.

**Figure A2:**
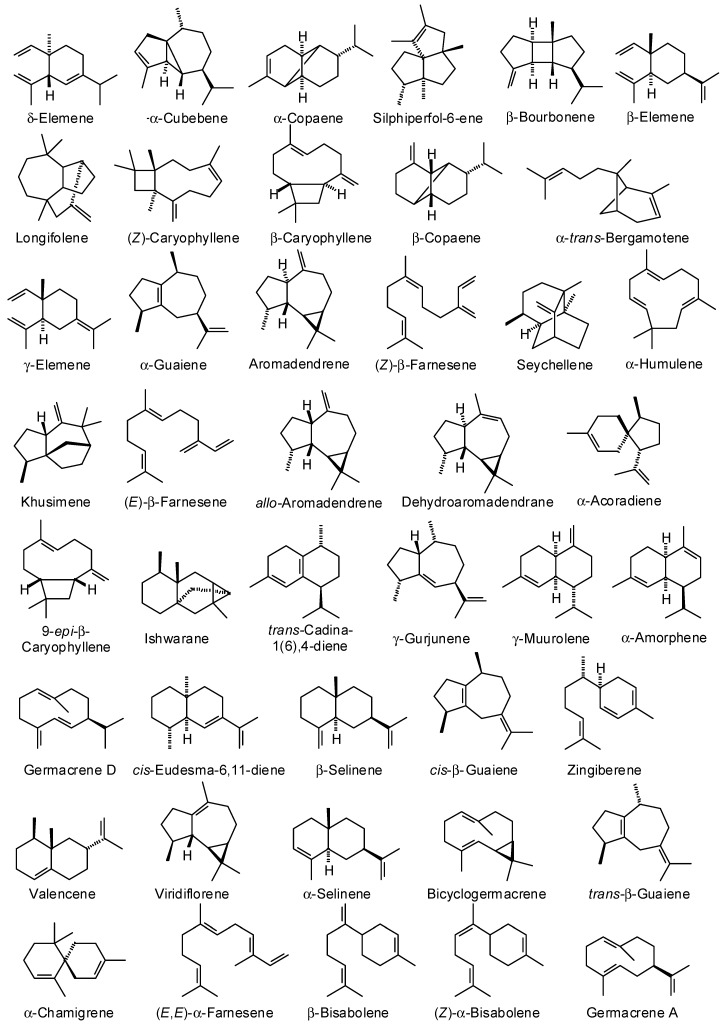

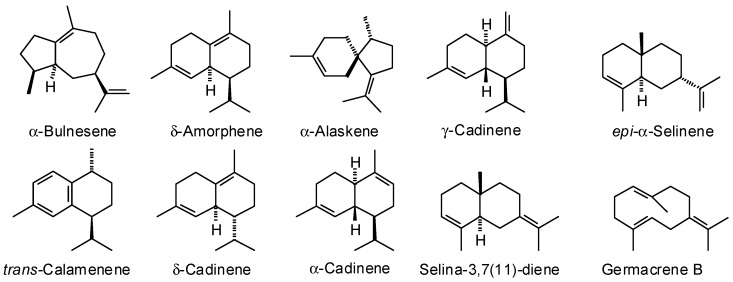
Major sesquiterpene hydrocarbons found in Neotropical *Piper* species.

**Figure A3:**
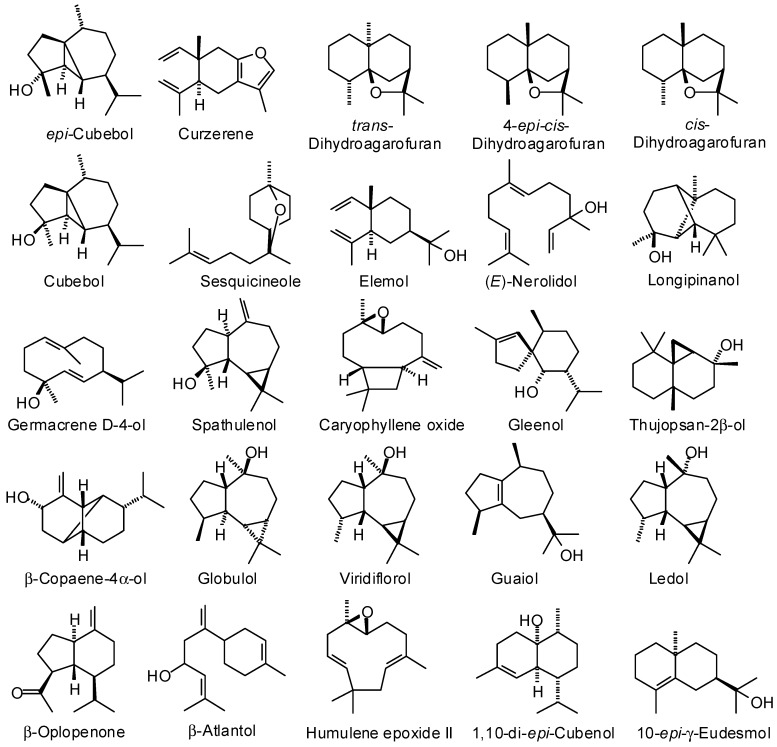

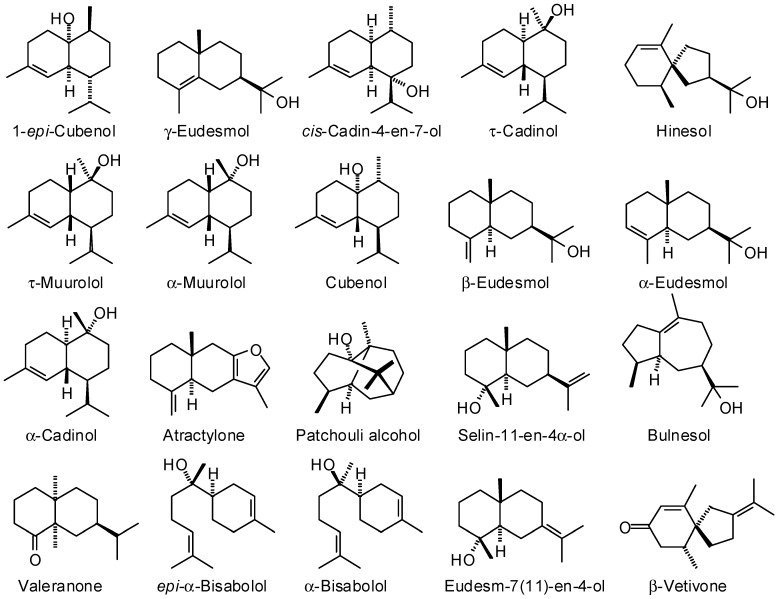
Major oxygenated sesquiterpenoids found in Neotropical *Piper* species.

**Figure A4:**
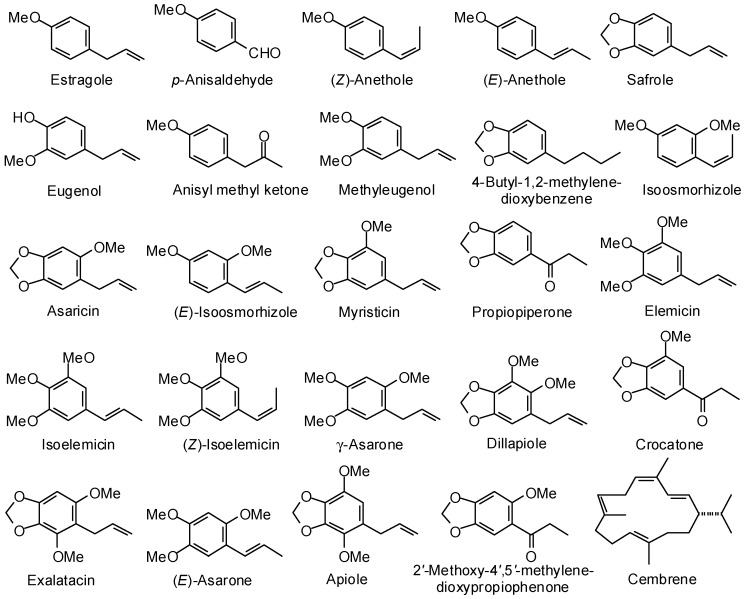
Major phenylpropanoids and miscellaneous compounds found in Neotropical *Piper* species.

## Abbreviations

ACh	Acetylcholine
AChE	Acetylcholine esterase
DL	Detection limit
DPPH	2,2-Diphenyl-1-picrylhydrazyl (radical)
EO	Essential oil
FIOCRUZ	Fundação Oswaldo Cruz (Oswaldo Cruz Foundation)
GC-MS	Gas chromatography-mass spectrometry
HD	Hydrodistillation
IC_50_	Median inhibitory concentration
ID_50_	Median inhibitory dose
LC_50_	Median lethal concentration
MFC	Minimum fungicidal concentration
MH	Monoterpene hydrocarbons
MIC	Minimum inhibitory concentration
MWHD	Microwave-assisted hydrodistillation
OM	Oxygenated monoterpenoids
OS	Oxygenated sesquiterpenoids
PCA	Principal component analysis
PP	Phenylpropanoids
RI	Retention index
SD	Steam distillation
SH	Sesquiterpene hydrocarbons
spp.	Species (plural)
TLC	Thin-layer chromatography

## Appendix A

**Table A1:** Neotropical *Piper* essential oil compositions and biological activities.

*Piper* species	Collection Site	Essential Oil	Major Components (>5%)	Bioactivity of EO	Ref.
*P. abutiloides* Kunth	Cultivated (State University of Campinas, São Paulo, Brazil)	Leaf (HD)	---	Antibacterial (*Escherichia coli*, MIC 700 μg/mL)	[52]
*P. acutifolium* Ruiz & Pav.	La Florida, Cajamarca, Peru	Leaf (HD)	(*E*)-β-Ocimene (8.1%), α-copaene (6.1%), β-caryophyllene (7.9%), *allo*-aromadendrene (6.0%), α-cadinene (6.7%), δ-cadinene (6.8%), dillapiole (5.9%)	---	[99]
*P. aduncum* L.	Serra do Navio, Amapá state, Brazil	Aerial parts (HD)	Limonene (5.2%), γ-terpinene (7.1%), terpinen-4-ol (11.0%), piperitone (15.1%), dillapiole (31.5%)	---	[15]
*P. aduncum* L.	Melgaço, Pará state, Brazil	Aerial parts (HD)	γ-Terpinene (6.5%), terpinen-4-ol (7.3%), piperitone (13.9%), dillapiole (50.8%)	---	[15]
*P. aduncum* L.	Benfica, Pará state, Brazil	Aerial parts (HD)	Piperitone (7.0%), dillapiole (56.3%)	---	[15]
*P. aduncum* L.	Belém, Pará state, Brazil	Aerial parts (HD)	Dillapiole (82.2%)	---	[15]
*P. aduncum* L.	Belém, Pará state, Brazil	Aerial parts (HD)	Dillapiole (86.9%)	---	[15]
*P. aduncum* L.	Manaus, Amazonas state, Brazil	Aerial parts (HD)	Dillapiole (91.1%)	---	[15]
*P. aduncum* L.	Manaus-Caracaraí, Amazonas, Brazil	Aerial parts (HD)	Dillapiole (97.3%)	---	[15]
*P. aduncum* L.	Cruzero do Sul, Acre state, Brazil	Aerial parts (HD)	Dillapiole (88.1%)	---	[15]
*P. aduncum* L.	Pinar del Río, Cuba	Leaf (HD)	Dillapiole (82.2%)	---	[27]
*P. aduncum* L.	Valle del Sajta, Cochabamba, Bolivia	Leaf (HD)	α-Pinene (9.0%), β-pinene (7.1%), limonene (5.0%), 1,8-cineole (40.5%), asaricin (12.9%)	---	[100]
*P. aduncum* L.	Altos de Campana National Park, Panama	Leaf (HD)	α-Pinene (8.8%), linalool (8.6%), β-caryophyllene (17.4%), aromadendrene (13.4%)	---	[100]
*P. aduncum* L.	Reserva da Ripasa, Ibaté, São Paulo state, Brazil	Leaf (HD)	(*E*)-β-Ocimene (5.0%), linalool (31.7%), β-caryophyllene (9.1%), α-humulene (5.5%), bicyclogermacrene (11.2%), (*E*)-nerolidol (10.4%)	Antifungal, TLC bioautography (*Cladosporium sphareospermum*)	[59]
*P. aduncum* L.	Reserva da Ripasa, Ibaté, São Paulo state, Brazil	Floral (HD) ^a^	α-Terpinene (6.8%), (*Z*)-β-ocimene (5.6%), (*E*)-β-ocimene (11.1%), γ-terpinene (12.0%), linalool (41.2%), (*E*)-nerolidol (6.1%)	---	[59]
*P. aduncum* L.	Reserva da Ripasa, Ibaté, São Paulo state, Brazil	Stem (HD)	α-Pinene (7.2%), β-pinene (14.2%), limonene (8.7%), (*Z*)-β-ocimene (5.5%), (*E*)-β-Ocimene (13.3%), linalool (11.8%), β-caryophyllene (7.6%), α-humulene (6.3%), (*E*)-nerolidol (10.6%)	Antifungal, TLC bioautography (*Cladosporium cladosporioides*, *Cladosporium sphareospermum*)	[59]
*P. aduncum* L.	Brejo da Madre de Deus, Matas Serranas, Pernambuco state, Brazil	Leaf (HD)	(*E*)-Nerolidol (80.6–82.5%), longipinanol (2.4–5.6%)	---	[26]
*P. aduncum* L.	Serra Negra, Matas Serranas, Pernambuco state, Brazil	Leaf (HD)	(*E*)-Nerolidol (79.2–81.2%), longipinanol (11.1–13.6%)	---	[26]
*P. aduncum* L.	Cultivated (State University of Campinas, São Paulo, Brazil)	Leaf (HD)	---	Antibacterial (*Escherichia coli*, MIC 500 μg/mL)	[52]
*P. aduncum* L.	Bulo Bulo, Bolivia	Leaf (SD)	α-Pinene (8.0–8.9%), β-pinene (6.6–7.0%), 1,8-cineole (42.0–42.5%), (*E*)-β-ocimene (6.4%), bicyclogermacrene (3.8–6.0%), asaricin (9.2–10.5%)	---	[101]
*P. aduncum* L.	Wasak'entsa reserve, Ecuador	Aerial parts (HD)	(*E*)-β-Ocimene (10.4%), piperitone (8.5%), dillapiole (45.9%)	Antifungal activity against dermatophytes (*Trichophyton mentagrophytes*, MIC 500 μg/mL, IC_50_ 92.7 μg/mL; *Trichophyton tonsurans*, MIC 500 μg/mL, IC_50_ 108.7 μg/mL; *Nantzzia cajetani*, IC_50_ 195 μg/mL)	[65]
*P. aduncum* L.	Ducke Reserve, Manaus, Amazonas state, Brazil	Leaf (HD)	Dillapiole (94.8%)	Acaricidal (*Rhipicephalus* (*Boophilus*) *microplus*, LC_50_ 9.3 mg/mL)	[28]
*P. aduncum* L.	Santo Antonio do Tauá, Pará state, Brazil	Aerial parts (HD)	Dillapiole (86.9%)	Larvicidal and insecticidal activity against mosquitoes (*Anopheles marajoara*, LC_50_ 50.9 μg/mL, 417 μg/mL, respectively; *Aedes aegypti*, LC_50_ 54.5 μg/mL, 401 μg/mL, respectively)	[16]
*P. aduncum* L.	Araraquara, São Paulo state, Brazil	Leaf (HD)	(*E*)-β-Ocimene (5.0%), linalool (31.8%), β-caryophyllene (9.3%), α-humulene (5.5%), bicyclogermacrene (11.3%), (*E*)-nerolidol (10.3%)	Antifungal, broth dilution assay (*Cryptococcus neoformans*, MIC 62.5 μg/mL)	[35]
*P. aduncum* L.	Brazlândia, Distrito Federal, Brazil	Leaf (HD)	β-Phellandrene (6.8%), γ-terpinene (8.3%), terpinen-4-ol (15.0%), piperitone (22.7%), asaricin (5.6%)	---	[16]
*P. aduncum* L.	Parque do Guará, Distrito Federal, Brazil	Leaf (HD)	β-Phellandrene (6.6%), γ-terpinene(8.2%), terpinen-4-ol (16.8%), piperitone (24.9%)	---	[16]
*P. aduncum* L.	Córrego Bananal, Distrito Federal, Brazil	Leaf (HD)	(*E*)-β-Ocimene (11.6%), terpinen-4-ol (6.7%), piperitone (11.0%), asaricin (15.8%)	---	[16]
*P. aduncum* L.	Fazenda Água Limpa, Distrito Federal, Brazil	Leaf (HD)	Piperitone (16.3%), dillapiole (49.5%)	---	[16]
*P. aduncum* L.	Mata de Dois Irmãos, Recife, Pernambuco, Brazil	Leaf (HD)	Dillapiole (79.0%)	---	[29]
*P. aduncum* L.	Belém, Pará state, Brazil	Aerial parts (HD)	Dillapiole (64.4%)	Insecticidal (*Solenopsis saevissima*, IC_50_ 135 μg/mL)	[41]
*P. aduncum* L.	Bocaiuva, Minas Gerais state, Brazil	Leaf (HD)	α-Pinene (14.2%), β-pinene (9.0%), 1,8-cineole (57.2%)	---	[25]
*P. aduncum* L.	Montes Claros, Minas Gerais state, Brazil	Leaf (HD)	(*E*)-β-Ocimene (13.4%), valencene (6.9%), (*E*)-nerolidol (5.9%)	---	[25]
*P. aduncum* L.	Topes de Collantes Nature Reserve, Escambray Mountains, Cuba	Leaf (HD)	Camphene (10.9%), 1,8-cineole (8.7%), camphor (17.1%), piperitone (34.0%), viridiflorol (7.4%)	Antioxidant (DPPH radical scavenging assay, IC_50_ 30.1 μg/mL)	[50]
*P. aduncum* L.	Gallery Forest, Angico River, Minas Gerais state, Brazil	Leaf (HD)	1,8-Cineole (55.8%), α-terpineol (5.9%)	Egg hatch inhibition (*Haemonchus contortus*, IC_50_ 2.6 mg/mL)	[102]
*P. aduncum* L.	Santo Antonio do Tauá, Pará state, Brazil	Aerial parts (HD)	Dillapiole (85.9%)	Antifungal activity against dermatophytes (*Trichophyton mentagrophytes*, MIC 500 μg/mL; *Epidermophyton floccosum*, MIC 500 μg/mL; *Microsporum canis*, MIC 250 μg/mL; Microsporum gypseum, MIC 250 μg/mL; Aspergillus fumigatus, MIC 3.9 μg/mL)	[30]
*P. aduncum* L.	Cultivated, Federal University of Lavras, Brazil	Leaf (HD)	Linalool (9.3–13.4%), β-caryophyllene (5.1–6.7%), α-humulene (8.5–10.6%), (*E*)-nerolidol (14.3–16.7%), spathulenol (0–5.6%), *cis*-cadin-4-en-7-ol (7.5–12.2%)	---	[103]
*P. aduncum* L.	Cultivated, Federal University of Lavras, Brazil	Root (HD)	α-Selinene (14.1–16.5%), geranyl 2-methylbutyrate (8.9–13.6%), bulnesol (4.6–6.1%), elemicin (4.6–5.9%), dillapiole (13.0–18.4%), apiole (16.3–29.5%)	---	[103]
*P. aduncum* L.	Monte Alegre do Sul, São Paulo state, Brazil	Leaf (HD)	α-Pinene (6.4%), safrole (13.3%), valencene (9.7%), spathulenol (10.6%), asaricin (14.9%)	---	[31]
*P. aduncum* L.	Votuporanga, São Paulo state, Brazil	Leaf (HD)	Safrole (10.8%), asaricin (80.1%)	---	[31]
*P. aduncum* L.	Votuporanga, São Paulo state, Brazil	Leaf (HD)	Safrole (10.5%), asaricin (73.4%)	---	[31]
*P. aduncum* L.	Belém, Pará state, Brazil	Aerial parts (HD)	Dillapiole (73.0%)	---	[62]
*P. aduncum* L.	Cerro Azul, Paraná state, Brazil	Leaf (HD)	(*Z*)-β-Ocimene (7.0%), (*E*)-β-ocimene (13.9%), safrole (6.2%), bicyclogermacrene (20.9%), γ-cadinene (5.5%), spathulenol (5.3%)	Antileishmanial (*L. amazonensis* promastigotes, IC_50_ 25.9 μg/mL; *L. amazonensis* axenic amastigotes, IC_50_ 36.2 μg/mL)	[70]
*P. aduncum* L.	Universidade Federal de Lavras, Matto Grosso state, Brazil	Leaf (HD)	Linalool (13.4%), (*E*)-nerolidol (25.2%), spathulenol (6.3%)	Antitrypanosomal (*T. cruzi* trypomastigotes, IC_50_ 2.8 μg/mL; linalool is the active agent, IC_50_ 0.31 μg/mL) Antileishmanial (*L. braziliensis* promatigotes, IC_50_ 77.9 μg/mL; (*E*)-nerolidol is the active agent, IC_50_ 74.3 μg/mL)	[67,68]
*P. aduncum* L.	Institute of Pharmacy and Food, Havana, Cuba	Aerial parts (HD)	Camphene (5.9%), camphor (17.1%) piperitone (23.7%), viridiflorol (14.5%)	Antiprotozoal (*Plasmodium falciparum*, IC_50_ 1.3 μg/mL; *Trypanosoma brucei*, IC_50_ 2.0 μg/mL; *Trypanosoma cruzi*, IC_50_ 2.1 μg/mL; *Leishmania amazonensis*, IC_50_ 23.8 μg/mL; *Leishmania donovani*, IC_50_ 7.7 μg/mL; *Leishmania infantum*, IC_50_ 8.1 μg/mL)	[32]
*P. aduncum* subsp. *ossanum* (C. DC.) Saralegui [syn. *P. ossanum* (C. DC.) Trel.]	Pinar del Río, Cuba	Leaf (HD)	Camphene (6.1%), camphor (8.3%), piperitone (12.9%), β-caryophyllene (6.7%), germacrene D (8.2%), 1-*epi*-cubenol (6.2%)	---	[104]
*P. aduncum* subsp. *ossanum* (C. DC.) Saralegui [syn. *P. ossanum* (C. DC.) Trel.]	Artemisa Province, Cuba	Leaf (HD)	Camphene (5.4–7.4%), camphor (9.4–13.9%), piperitone (19.0–20.1%), viridiflorol (13.0–18.8%)	Antiprotozoal (*Plasmodium falciparum*, IC_50_ 1.5 μg/mL; *Trypanosoma brucei*, IC_50_ 8.1 μg/mL; *Trypanosoma cruzi*, IC_50_ 8.0 μg/mL; *Leishmania amazonensis*, IC_50_ 19.3 μg/mL; *Leishmania infantum*, IC_50_ 32.5 μg/mL), antibacterial (*Staphylococcus aureus*, IC_50_ 39.5 μg/mL)	[53]
*P. aequale* Vahl	Monteverde, Costa Rica	Leaf (HD)	α-Pinene (39.3%), sabinene (18.4%), limonene (6.7%)	Antibacterial (*Bacillus cereus*, MIC 156 μg/mL)	[34]
*P. aequale* Vahl	Carajás National Forest, Parauapebas, Pará state, Brazil	Aerial parts (HD)	α-Pinene (12.6%), β-pinene (15.6%), δ-elemene (19.0%), bicyclogermacrene (5.5%), cubebol (7.2%), β-atlantol (5.9%)	Cytotoxic (HCT-116 human colorectal carcinoma, IC_50_ 8.69 μg/mL; ACP03 human gastric adenocarcinoma, IC_50_ 1.54 μg/mL; essential oil induced apoptosis in ACP03 cells)	[95]
*P.* sp. aff. *aereum*	Monteverde, Costa Rica	Leaf (HD)	β-Caryophyllene (6.6%), δ-cadinene (7.3%), guaiol (41.2%), α-muurolol (5.8%), α-cadinol (9.2%)	Antibacterial (*Bacillus cereus*, MIC 78 μg/mL; *Staphylococcus aureus*, MIC 78 μg/mL), cytotoxic (MCF-7 human breast adenocarcinoma)	[34]
*P. aleyreanum* C. DC.	Porto Velho, Rondônia state, Brazil	Leaf (HD)	α-Pinene (7.0%), β-pinene (14.4%), α-phellandrene (8.6%), (*Z*)-caryophyllene (17.5%), β-caryophyllene (18.6%), δ-cadinene (6.2%)	---	[105]
*P. aleyreanum* C. DC.	Porto Velho, Rondônia state, Brazil	Aerial parts (HD)	Camphene (5.2%), β-pinene (9.0%), spathulenol (6.7%), caryophyllene oxide (11.5%)	Antinociceptive, anti-inflammatory (mouse model)	[90]
*P. aleyreanum* C. DC.	Carajás National Forest, Parauapebas, Pará state, Brazil	Aerial parts (HD)	δ-Elemene (8.2%), β-elemene (16.3%), β-caryophyllene (6.2%), germacrene D (6.9%), bicyclogermacrene (9.2%), spathulenol (5.2%)	Antifungal, TLC bioautography (*Cladosporium cladosporioides*, *Cladosporium sphaerospermum*), cytotoxic (SKMel19 human melanoma, IC_50_ 7.4 μg/mL)	[61]
*P. amalago* L.	Fazenda Sucupira, Embrapa, Brasília, Brazil	Leaf (HD)	α-Pinene (30.5%), camphene (8.9%), limonene (6.8%), borneol (5.7%)	---	[33]
*P. amalago* L.	Monteverde, Costa Rica	Leaf (HD)	α-Phellandrene (1.7–8.1%), β-elemene (11.5–24.6%), β-caryophyllene (15.9–23.3%), germacrene D (28.9–29.4%), germacrene A (6.5–9.7%)	---	[34]
*P. amalago* L.	Morro Reuter, Rio Grande do Sul state, Brazil	Aerial parts (HD)	α-Pinene (5.2%), limonene (20.5%), δ-elemene (6.8%), zingiberene (11.2%)	---	[106]
*P. amalago* L.	Universidade de São Paulo, Brazil	Leaf (HD)	γ-Muurolene (7.3%), germacrene D (9.9%), bicyclogermacrene (27.9%), spathulenol (19.2%), α-cadinol (7.6%)	---	[35]
*P. amalago* L.	Dourados, Mato Grosso do Sul, Brazil	Leaf (HD)	*p*-Cymene (9.4%), methyl geranate (7.8%), α-amorphene (25.7%), cubenol (6.2%)	---	[107]
*P. amalago* L.	Dourados, Mato Grosso do Sul, Brazil	Stem (HD)	Longifolene (6.6%), α-amorphene (23.3%), α-muurolol (9.3%)	---	[107]
*P. amalago* L.	Dourados, Mato Grosso do Sul, Brazil	Root (HD)	α-Amorphene (14.4%)	---	[107]
*P. amalago* L.	Dourados, Mato Grosso do Sul, Brazil	Floral (HD) ^a^	*p*-Cymene (9.3%), limonene (10.5%), silphiperfol-6-ene (13.5%), *allo*-aromadendrene (18.5%), α-muurolol (5.0%)	---	[107]
*P. amalago* L.	Campinas, São Paulo state, Brazil	Leaf (HD)	α-Pinene (14.8%), β-phellandrene (39.3%), germacrene D (11.7%)	---	[31]
*P. amalago* L.	Campinas, São Paulo state, Brazil	Leaf (HD)	α-Pinene (6.7%), sabinene (6.7%), β-phellandrene (15.9%), bicyclogermacrene (20.8%), spathulenol (9.1%)	---	[31]
*P. amalago* L.	Campinas, São Paulo state, Brazil	Leaf (HD)	α-Pinene (11.7%), β-phellandrene (33.1%), bicyclogermacrene (15.0%)	---	[31]
*P. amalago* L.	Adamantina, São Paulo state, Brazil	Leaf (HD)	Sabinene (8.2%), myrcene (6.8%), β-phellandrene (12.3%), bicyclogermacrene (19.4%), γ-muurolene (5.9%), spathulenol (5.6%)	---	[31]
*P. amalago* var. *medium* (Jacq.) Yunck.	Fênix, Paraná state, Brazil	Floral (HD) ^a^	β-Phellandrene (7.3–8.2%), bicyclogermacrene (3.0–9.1%), δ-cadinene (2.3–6.6%), (*E*)-nerolidol (14.2–19.9%), germacrene D-4-ol (10.3–12.7%), τ-cadinol (4.9–6.1%), α-cadinol (8.2–11.1%)	---	[108]
*P. amplum* Kunth	Pariquera-Açu, São Paulo state, Brazil	Leaf (HD)	α-Pinene (18.1%), (*Z*)-β-ocimene (10.5%), limonene (8.6%), β-caryophyllene (8.8%), germacrene D (5.5%)	---	[31]
*P. angustifolium* Lam.	Cuzco, Peru	Aerial parts (HD)	Camphene (22.4%), camphor (25.3%), isoborneol (12.8%)	Antibacterial, broth dilution assay (*Pseudomonas aeruginosa*, MIC 30 μg/mL; *Escherichia coli*, MIC 100 μg/mL); antifungal, broth dilution assay (*Trichophyton mentagrophytes*, MIC 10 μg/mL; *Candida albicans*, MIC 50 μg/mL; *Cryptococcus neoformans*, MIC 50 μg/mL; *Aspergillus flavus*, MIC 100 μg/mL)	[109]
*P. angustifolium* Lam.	Abobral Subregion of the Pantanal of Mato Grosso do Sul, Brazil	Leaf (HD)	α-Pinene (5.9%), (*E*)-nerolidol (5.8%), spathulenol (23.8%), caryophyllene oxide (13.1%)	Antileishmanial (*L. infantum* amastigotes, IC_50_ 1.43 μg/mL)	[69]
*P. anonifolium* Kunth	Bujaru, Pará state, Brazil	Aerial parts (HD)	α-Pinene (41.1–45.7%), β-pinene (17.2–18.6%), limonene (6.1–8.5%), β-caryophyllene (2.5–6.3%)	---	[110]
*P. anonifolium* Kunth	Santa Isabel, Pará state, Brazil	Aerial parts (HD)	α-Pinene (53.1%), β-pinene (22.9%)	---	[110]
*P. anonifolium* Kunth	Ananindeua, Pará state, Brazil	Aerial parts (HD)	α-Pinene (7.3%), limonene (5.9%), ishwarane (19.1%), germacrene D (9.6%), α-eudesmol (33.5%)	---	[110]
*P. anonifolium* Kunth	Carajás National Forest, Parauapebas, Pará state, Brazil	Aerial parts (HD)	α-Pinene (8.8%), β-selinene (12.7%), α-selinene (11.9%), selin-11-en-4α-ol (20.0%)	Antifungal, TLC bioautography (*Cladosporium cladosporioides*, *Cladosporium sphaerospermum*); enzyme inhibitory, TLC bioautography (acetylcholinesterase)	[61]
*P. arboreum* Aubl.	Chepo, Panama	Leaf (HD)	β-Pinene (6.6%), α-copaene (7.4%), germacrene D (5.3%), δ-cadinene (25.8%), (*E*)-nerolidol (5.2%)	---	[111]
*P. arboreum* Aubl.	Fazenda Sucupira, Embrapa, Brasília, Brazil	Leaf (HD)	Bicyclogermacrene (12.1%), spathulenol (8.4%), caryophyllene oxide (10.2%) ^b^	---	[33]
*P. arboreum* Aubl.	Universidade Estadual Paulista, Araraquara, São Paulo state, Brazil	Leaf (HD)	β-Caryophyllene (25.1%), germacrene D (9.6%), bicyclogermacrene (49.5%)	---	[59]
*P. arboreum* Aubl.	Universidade Estadual Paulista, Araraquara, São Paulo state, Brazil	Floral (HD) ^a^	Limonene (6.3%), linalool (10.4%), β-elemene (5.3%), β-caryophyllene (6.6%), germacrene D (49.3%), germacrene A (8.5%)	---	[59]
*P. arboreum* Aubl.	Universidade Estadual Paulista, Araraquara, São Paulo state, Brazil	Stem (HD)	δ-3-Carene (18.7%), α-copaene (9.0%), β-caryophyllene (26.5%), bicyclogermacrene (21.1%)	---	[59]
*P. arboreum* Aubl.	Antonina, Paraná state, Brazil	Leaf (HD)	α-Copaene (5.6%), β-caryophyllene (12.6%), *trans*-cadina-1(6),4-diene (9.6%), spathulenol (7.9%), caryophyllene oxide (5.9%), 1-*epi*-cubenol (10.4%), α-cadinol (5.4%)	Antileishmanial (*L. amazonensis* promastigotes, IC_50_ 15.2 μg/mL; *L. amazonensis* axenic amastigotes, IC_50_ > 200 μg/mL)	[70]
*P. arboreum* var. *latifolium* (C. DC.) Yunck.	Rondônia state, Brazil	Leaf (SD)	Octanal (5.5%), germacrene D (72.9%), γ-elemene (6.8%) ^c^	---	[112]
*P. artanthe* C. DC.	San Migues, Santander, Colombia	Aerial parts (HD)	δ-Elemene (11.7%), β-caryophyllene (10.2%), *epi*-cubebol (8.9%), cubebol (6.3%), myristicin (6.4%), apiole (14.5%)	---	[113]
*P. augustum* Rudge	Reserva Biológica Alberto Manuel Brenes, Costa Rica	Leaf (HD)	α-Pinene (10.5%), α-phellandrene (14.7%), limonene (13.0%), β-phellandrene (5.6%), linalool (10.3%), β-caryophyllene (13.5%)	---	[114]
*P. augustum* Rudge	Valle de Anton, Cerro Caracoral, Cocle, Panama	Leaf (HD)	α-Pinene (6.0%), β-elemene (12.3%), cembrene (11.7%), cembratrienol 1 (25.4%), cembratrienol 2 (8.6%)	---	[47]
*P. auritum* Kunth	Boca de Uracillo, Colon Province, Panama	Leaf (HD)	Safrole (70%)	---	[115]
*P. auritum* Kunth	Güira de Melena, Cuba	Leaf (HD)	Safrole (64.5%)	---	[116]
*P. auritum* Kunth	Monteverde, Costa Rica	Floral (HD) ^a^	Safrole (93.2%)	---	[49]
*P. auritum* Kunth	Universidad de La Habana, Cuba	Aerial parts (HD)	Safrole (86.9%)	Antileishmanial (promastigotes of *L. major*, IC_50_ 29.1 μg/mL; *L. mexicana*, IC_50_ 63.3 μg/mL; *L. braziliensis*, IC_50_ 52.1 μg/mL; *L. donovani*, IC_50_ 12.8 μg/mL; amastigotes of *L. donovani*, IC_50_ 22.3 μg/mL)	[117]
*P. auritum* Kunth	Cali, Valle del Cauca, Colombia	Aerial parts (MWHD)	Safrole (91.3%)	---	[118]
*P. auritum* Kunth	Topes de Collantes Nature Reserve, Escambray Mountains, Cuba	Leaf (HD)	Camphene (5.5%), safrole (71.8%)	Antioxidant (DPPH radical scavenging assay, IC_50_ 14.8 μg/mL)	[50]
*P. barbatum* Kunth	Amazonas region, Peru	Aerial parts (HD)	Crocatone (10.9%), (*E*)-asarone (14.1%), apiole (8.0%), 2′-methoxy-4′,5′-methylenedioxy-propiophenone (29.5%)	---	[119]
*P. biasperatum* Trel.	Monteverde, Costa Rica	Leaf (HD)	β-Elemene (46.4%), germacrene D (9.5%), bicyclogermacrene (14.1%), germacrene A (13.2%)	Cytotoxic (MCF-7 human breast adenocarcinoma)	[34]
*P. bogotense* C. DC.	Ipiales, Nariño, Colombia	Aerial parts (MWHD)	α-Pinene (8.7%), α-phellandrene (13.7%), limonene (5.3%), *trans*-sabinene hydrate (14.2%)	Antitrypanosomal (*T. cruzi* epimastigotes, IC_50_ 10.1 μg/mL); cytotoxic (Vero cells, IC_50_ 90.1 μg/mL), Antifungal, broth dilution assay (*Trichophyton rubrum*, MIC 79 μg/mL; *Trichophyton mentagrophytes*, MIC 500 μg/mL); cytotoxic (Vero cells, IC_50_ 25.8 μg/mL)	[118,120,121]
*P. brachypodon* (Benth.) C. DC.	Quibdó, Chocó, Colombia	Aerial parts (MWHD)	β-Caryophyllene (20.2%), 9-*epi*-β-caryophyllene (5.8%), germacrene D (5.9%), bicyclogermacrene (8.1%), spathulenol (5.7%), caryophyllene oxide (10.8%)	---	[120]
*P. brachypodon* (Benth.) C. DC.	Tutunendo, Chocó, Colombia	Aerial parts (MWHD)	β-Caryophyllene (20.2%), 9-*epi*-(*E*)-caryophyllene (5.8%), germacrene D (5.9%), bicyclogermacrene (8.1%), spathulenol (5.7%), caryophyllene oxide (10.8%)	Antiprotozoal (*Trypanosoma cruzi* epimastigotes, IC_50_ 0.34 μg/mL; *Leishmania infantum* promastigotes, IC_50_ 23.4 μg/mL); cytotoxic (Vero cells, IC_50_ 30.5 μg/mL; THP-1 human monocytic leukemia, IC_50_ 66.3 μg/mL)	[118]
*P. brachypodon* var. *hirsuticaule* Yunck.	Samurindó, Chocó, Colombia	Aerial parts (MWHD)	β-Elemene (6.4%), β-caryophyllene (9.8%), α-guaiene (5.9%), germacrene D (16.7%), bicyclogermacrene (6.2%)	Antiprotozoal (*Trypanosoma cruzi* epimastigotes, IC_50_ 32.5 μg/mL; *Leishmania infantum* promastigotes, IC_50_ 93.6 μg/mL); cytotoxic (Vero cells, IC_50_ 86.4 μg/mL)	[118]
*P. bredemeyeri* Jacq.	Monteverde, Costa Rica	Leaf (HD)	β-Elemene (34.0%), β-caryophyllene (24.2%), germacrene D (21.7%), bicyclogermacrene (14.1%), germacrene A (11.4%)	Antibacterial, broth dilution assay (*Bacillus cereus*, MIC 78 μg/mL), enzyme inhibitory (cruzain, IC_50_ 0.96 μg/mL)	[34]
*P. bredemeyeri* Jacq.	Pueblo Bello, Cesar, Colombia	Aerial parts (MWHD)	α-Pinene (20.3%), β-pinene (32.3%), β-caryophyllene (6.3%)	Antifungal, broth dilution assay (*Trichophyton rubrum*, MIC 157 μg/mL; *Trichophyton mentagrophytes*, MIC 125 μg/mL); cytotoxic (Vero cells, IC_50_ 15.2 μg/mL)	[121]
*P. caldense* C. DC.	Recife, Pernambuco state, Brazil	Leaf (HD)	δ-Cadinene (5.6%), thujopsan-2β-ol (7.4%), α-muurolol (9.0%), α-cadinol (19.0%)	Antibacterial, agar diffusion assay (*Bacillus subtilis*, *Staphylococcus aureus*, *Klebsiella pneumoniae*)	[54]
*P. caldense* C. DC.	Recife, Pernambuco state, Brazil	Root (HD)	Valencene (10.5%), pentadecane (35.7%), selina-3,7(11)-diene (5.4%)	Antibacterial, agar diffusion assay (*Bacillus subtilis*, *Staphylococcus aureus*, *Escherichia coli*, *Klebsiella pneumoniae*, *Pseudomonas aeruginosa*)	[54]
*P. caldense* C. DC.	Recife, Pernambuco state, Brazil	Stem (HD)	Terpinen-4-ol (18.5%), α-terpineol (15.3%), caryophyllene oxide (6.2%), α-cadinol (9.8%)	Antibacterial, agar diffusion assay (*Bacillus subtilis*, *Pseudomonas aeruginosa*)	[54]
*P. callosum* Ruiz & Pav.	Marituba, Pará state, Brazil	Aerial parts (HD)	Safrole (69.2%), methyleugenol (8.6%)	Insecticidal (*Solenopsis saevissima*, IC_50_ > 500 μg/mL)	[41]
*P. callosum* Ruiz & Pav.	Barcarena, Pará state, Brazil	Aerial parts (HD)	Safrole (66.0%), methyl eugenol (10.2%)	Enzyme inhibitory (acetylcholinesterase)	[62]
*P. carniconnectivum* C. DC.	Porto Velho, Rondônia state, Brazil	Leaf (HD)	β-Pinene (6.3%), caryophyllene oxide (21.3%)	---	[122]
*P. carniconnectivum* C. DC.	Porto Velho, Rondônia state, Brazil	Stem (HD)	α-Pinene (8.0%), β-pinene (19.0%), spathulenol (23.7%), caryophyllene oxide (7.8%)	---	[122]
*P. carpunya* Ruiz & Pav.	Cajamarca region, Peru	Leaf (HD)	α-Terpinene (12.1%), *p*-cymene (10.9%), 1,8-cineole (13.0%), safrole (14.9%), bicyclogermacrene (6.7%), spathulenol (9.8%)	---	[123]
*P. carpunya* Ruiz & Pav.	Cajamarca region, Peru	Floral (HD) ^a^	α-Pinene (6.2%), α-terpinene (9.8%), *p*-cymene (7.7%), 1,8-cineole (30.2%), safrole (32.0%)	---	[123]
*P. cernuum* Vell.	Universidade de São Paulo, Brazil	Leaf (HD)	α-Pinene (7.2%), β-pinene (6.2%), β-caryophyllene (20.7%), germacrene D (6.7%), bicyclogermacrene (21.9%)	Antimicrobial, agar diffusion assay (*Staphylococcus aureus*, *Candida albicans*)	[38]
*P. cernuum* Vell.	São Francisco de Assis Natural Reserve, Blumenau, Santa Catarina state, Brazil	Aerial parts (HD)	*trans*-Dihydroagarofuran (31.0%), elemol (12.0%), 10-*epi*-γ-eudesmol (13.0%)	---	[124]
*P. cernuum* Vell.	Universidade de São Paulo, Brazil	Leaf (HD)	β-Elemene (7.2%), β-caryophyllene (22.2%), germacrene D (9.3%), bicyclogermacrene (25.1%), (*Z*)-α-bisabolene (5.7%), spathulenol (7.2%)	---	[35,60]
*P. cernuum* Vell.	Universidade de São Paulo, Brazil	Floral (HD) ^a^	α-Copaene (6.5%), β-caryophyllene (9.8%), germacrene D (14.3%), bicyclogermacrene (6.5%), spathulenol (9.7%)	Antifungal, TLC bioautography (*Cladosporium cladosporioides*, *C. sphaerospermum*)	[60]
*P. cernuum* Vell.	Reserva da Matinha, Ilhéus, Bahia state, Brazil	Leaf (HD)	β-Elemene (11.6%), β-caryophyllene (8.3%), *cis*-β-guaiene (8.2%), γ-muurolene (7.6%), *epi*-cubebol (13.1%), spathulenol (9.6%), caryophyllene oxide (7.7%), valeranone (9.1%)	---	[125]
*P. cernuum* Vell.	Parque Ecológico do Pereque, Cubatão, São Paulo state, Brazil	Leaf (HD)	β-Elemene (30.0%), β-caryophyllene (16.3%), germacrene D (12.7%), bicyclogermacrene (19.9%)	Cytotoxic (B16F10-Nex2 murine melanoma, IC_50_ 30 μg/mL; A2058 human melanoma, IC_50_ 24 μg/mL; U87-MG human glioblastoma, IC_50_ 19.1 μg/mL; HeLa human cervical tumor, IC_50_ 23 μg/mL; HL-60 humal myloid leukemia, IC_50_ 16 μg/mL)	[39]
*P. cernuum* Vell.	Parque Ecológico do Pereque, Cubatão, São Paulo state, Brazil	Branches (HD)	Camphene (46.4%), *p*-cymene (5.8%), linalool (8.7%), α-terpineol (11.6%), carvacrol (11.6%) ^d^	Cytotoxic (B16F10-Nex2 murine melanoma, IC_50_ 39.0 μg/mL; A2058 human melanoma, IC_50_ 24.6 μg/mL; U87-MG human glioblastoma, IC_50_ 19.0 μg/mL; HeLa human cervical tumor, IC_50_ 23.6 μg/mL; HL-60 humal myloid leukemia, IC_50_ 15.5 μg/mL)	[96]
*P. cernuum* Vell.	Ubatuba, São Paulo state, Brazil	Leaf (HD)	α-Pinene (10.0%), camphene (6.3%), *trans*-dihydroagarofuran (28.7%), 10-*epi*-γ-eudesmol (13.5%), 4-*epi-cis*-dihydroagarofuran (10.8%)	---	[31]
*P. cernuum* Vell.	Pariquera-Açu, São Paulo state, Brazil	Leaf (HD)	α-Pinene (11.8%), camphene (8.7%), *trans*-dihydroagarofuran (33.8%), 10-*epi*-γ-eudesmol (12.2%)	---	[31]
*P. cernuum* Vell.	Antonina, Paraná state, Brazil	Leaf (HD)	α-Pinene (11.4%), β-pinene (7.9%), β-elemene (10.1%), β-caryophyllene (6.9%), spathulenol (11.5%), caryophyllene oxide (5.1%), τ-muurolol (6.2%), α-muurolol (5.8%)	Antileishmanial (*L. amazonensis* promastigotes, IC_50_ 27.1 μg/mL; *L. amazonensis* axenic amastigotes, IC_50_ > 200 μg/mL), anti-*Mycobacterium tuberculosis* (MIC 125 μg/mL)	[70]
*P. cernuum* Vell.	Blumenau, Santa Catarina state, Brazil	Leaf (HD)	α-Pinene (2.6–5.4%), β-caryophyllene (5.9–8.7%), 4-*epi-cis*-dihydroagarofuran (11.2–13.4%), *trans*-dihydroagarofuran (30.0–36.7%), elemol (5.9–9.2%), γ-eudesmol (8.3–13.3%)	Antibacterial, agar dilution assay (*Bacillus subtilis*, MIC 48 μg/mL; *Staphylococcus aureus*, MIC 780 μg/mL; *Streptococcus pyogenes*, MIC 780 μg/mL); antifungal, agar dilution assay (*Microsporum canis*, *Microsporum gypseum*, *Trichophyton mentagrophytes*, *Trichophyton rubrum*, *Epidermophyton flocosum*, *Cryptococcus neoformans*, MIC 48 μg/mL)	[37]
*P. cernuum* Vell. var. *cernuum*	Tijuca Forest, Rio de Janeiro state, Brazil	Leaf (HD)	α-Pinene (10.2%), camphene (5.3%), β-pinene (7.4%), *cis*-dihydroagarofuran (32.3%), elemol (6.7%)	---	[36]
*P. claussenianum* (Miq.) C. DC.	São Manoel, Castelo, Espírito Santo, Brazil	Leaf (HD)	Linalool (2.1–5.2%), (*E*)-nerolidol (81.4–83.3%)	Antileishmanial (promastigotes of *L. amazonensis*, IC_50_ 30.24 μg/mL) Anticandidal (*C. albicans*, MIC 0.2–1.26%)	[71,126]
*P. claussenianum* (Miq.) C. DC.	São Manoel, Castelo, Espírito Santo, Brazil	Floral (HD) ^a^	Linalool (50.2–54.5%), (*E*)-nerolidol (22.7–24.3%)	Antileishmanial (promastigotes of *L. amazonensis*, IC_50_ 1328 μg/mL) Anticandidal (*C. albicans*, MIC 0.04–0.1%) Antiparasitic (*Plasmodium falciparum* W2, IC_50_ 7.9 μg/mL)	[71,73,126]
*P. corcovadense* (Miq.) C. DC.	Jardim Botânico de Recife, Pernambuco, Brazil	Leaf (HD)	α-Pinene (5.9%), terpinolene (17.4%), 4-butyl-1,2-methylenedioxybenzene (30.6%), β-caryophyllene (6.3%)	Mosquito larvicidal activity (*Aedes aegypti*, LC_50_ 30.5 μg/mL)	[127]
*P. corrugatum* Kuntze	Valle de Anton, Cerro Caracoral, Cocle, Panama	Leaf (HD)	α-Pinene (12.2%), β-pinene (26.6%), limonene (8.2%), *p*-cymene (8.6%), 1,8-cineole (5.9%), (*E*)-nerolidol (12.8%), caryophyllene oxide (8.5%)	---	[47]
*P. crassinervium* Kunth	Universidade de São Paulo, Brazil	Leaf (HD)	β-Caryophyllene (8.1%), germacrene D (14.0%), bicyclogermacrene (9.2%), *epi*-α-selinene (5.0%), (*E*)-nerolidol (8.2%), spathulenol (9.8%), guiaol (5.8%), β-eudesmol (10.1%)	Antifungal, TLC bioautography (*Cladosporium cladosporioides*, *Cladosporium sphaerospermum*)	[35,60]
*P. crassinervium* Kunth	Mococa, São Paulo state, Brazil	Leaf (HD)	α-Pinene (11.5%), β-pinene (11.6%), β-caryophyllene (7.8%), germacrene D (9.2%), bicyclogermacrene (5.1%), guaiol (5.5%)	---	[31]
*P. curtispicum* C. DC.	Altos de Campana, Panama	Leaf (HD)	α-Pinene (19.4%), limonene (8.1%), β-caryophyllene (13.9%)	---	[47]
*P. cyrtopodon* C. DC.	Marituba, Pará state, Brazil	Aerial parts (HD)	α-Cubebene (5.1%), β-caryophyllene (19.2%), germacrene D (10.0%), bicyclogermacrene (13.0%), spathulenol (8.4%)	---	[128]
*P. cyrtopodon* C. DC.	Santarém, Pará state, Brazil	Aerial parts (HD)	*p*-Cymene (6.3%), germacrene D (17.9%), bicyclogermacrene (23.3%), (*E*)-nerolidol (6.6%), spathulenol (6.9%)	---	[128]
*P. cyrtopodon* C. DC.	Ananindeua, Pará state, Brazil	Aerial parts (HD)	α-Pinene (7.5%), β-pinene (6.0%), β-caryophyllene (34.6%), germacrene D (13.6%), bicyclogermacrene (21.4%), spathulenol (8.4%)	---	[128]
*P. cyrtopodon* C. DC.	Ananindeua, Pará state, Brazil	Aerial parts (HD)	β-Caryophyllene (18.8%), germacrene D (14.8%), bicyclogermacrene (14.0%), germacrene B (26.8%)	---	[128]
*P. cyrtopodon* C. DC.	Bujaru, Pará state, Brazil	Aerial parts (HD)	α-Cubebene (6.7%), β-caryophyllene (18.1%), germacrene D (13.6%), bicyclogermacrene (14.9%), germacrene B (10.1%)	---	[128]
*P. cyrtopodon* C. DC.	Manaus, Amazonas state, Brazil	Aerial parts (HD)	Germacrene D (7.5%), bicyclogermacrene (8.3%), α-cadinol (9.5%), *epi*-α-bisabolol (26.3%)	---	[128]
*P. dactylostigmum* Yunck.	Itacoatiara, Amazonas State, Brazil	Aerial parts (HD)	β-Caryophyllene (8.9%), γ-muurolene (5.9%), β-selinene (9.0%), α-selinene (8.0%), caryophyllene oxide (6.0%), τ-muurolol (7.5%), α-cadinol (21.7%)	---	[129]
*P. darienense* C. DC.	Parque Nacional Chagres, Panama	Leaf (HD)	Limonene (6.3%), (*E*)-β-farnesene (63.7%)	---	[47]
*P. demeraranum* (Miq.) C. DC.	Belém, Pará state, Brazil	Aerial parts (HD)	α-Pinene (7.3%), sabinene (12.9%), β-pinene (7.7%), limonene (20.2%)	---	[130]
*P. demeraranum* (Miq.) C. DC.	Ananindeua, Pará state, Brazil	Aerial parts (HD)	α-Pinene (6.1–12.3%), sabinene (17.0–22.7%), β-pinene (8.2–14.4%), limonene (30.6–40.3%)	---	[130]
*P. demeraranum* (Miq.) C. DC.	Adolpho Ducke Reserve, Manaus, Amazonas state, Brazil	Leaf (HD)	β-Pinene (6.7%), limonene (19.3%), β-elemene (33.1%), β-caryophyllene (6.0%), germacrene D (5.2%), β-selinene (5.0%), bicyclogermacrene (8.8%)	Antileishmanial (*L. amazonensis* promastigotes, IC_50_ 86.0 μg/mL; *L. amazonensis* amastigotes, IC_50_ 78.0 μg/mL; *L. guyanensis* promastigotes, IC_50_ 22.7 μg/mL)	[72]
*P. dilatatum* Rich.	Fazenda Sucupira, Embrapa, Brasília, Brazil	Leaf (HD)	(*E*)-β-Ocimene (19.7%), β-caryophyllene (11.4%), germacrene D (8.9%), bicyclogermacrene (8.8%), spathulenol (6.5%), caryophyllene oxide (5.3%)	---	[33]
*P. dilatatum* Rich.	Alto Alegre, Roraima state, Brazil	Aerial parts (HD)	β-Caryophyllene (11.7%), germacrene D (6.7%), α-selinene (6.1%), δ-cadinene (5.4%), caryophyllene oxide (6.1%), α-cadinol (12.2%)	---	[131]
*P. dilatatum* Rich.	Alto Alegre, Roraima state, Brazil	Aerial parts (HD)	β-Caryophyllene (15.5%), germacrene D (10.2%), α-selinene (6.9%), δ-cadinene (8.5%), hinesol (6.4%), α-cadinol (7.0%)	---	[131]
*P. dilatatum* Rich.	Benfica, Pará state, Brazil	Aerial parts (HD)	Germacrene D (12.6%), bicyclogermacrene (7.4%), (*E*)-nerolidol (10.2%), spathulenol (11.8%), hinesol (6.4%), α-cadinol (5.8%)	---	[131]
*P. dilatatum* Rich.	Belterra, Pará state, Brazil	Aerial parts (HD)	α-Pinene (9.7%), β-pinene (14.8%), (*Z*)-β-ocimene (10.0%), β-caryophyllene (7.4%), bicyclogermacrene (27.6%), spathulenol (15.0%)	---	[131]
*P. dilatatum* Rich.	Marituba, Pará state, Brazil	Aerial parts (HD)	*p*-Cymene (11.7%), β-selinene (6.4%), curzerene (13.8%), (*E*)-nerolidol (5.7%), α-eudesmol (8.0%), atractylone (5.1%)	---	[131]
*P. dilatatum* Rich.	Marituba, Pará state, Brazil	Aerial parts (HD)	Germacrene D (30.2%), bicyclogermacrene (9.4%), spathulenol (40.6%), hinesol (6.4%), α-cadinol (5.8%)	---	[131]
*P. dilatatum* Rich.	Serra dos Carajás, Pará state, Brazil	Aerial parts (HD)	*p*-Cymene (5.1%), β-elemene (21.8%), β-caryophyllene (5.1%), germacrene D (18.5%)	---	[131]
*P. dilatatum* Rich.	Serra dos Carajás, Pará state, Brazil	Aerial parts (HD)	β-Pinene (10.5%), limonene (6.4%), δ-elemene (7.6%), β-elemene (13.8%), bicyclogermacrene (7.9%), spathulenol (9.3%)	---	[131]
*P. dilatatum* Rich.	Angico, Tocantins state, Brazil	Aerial parts (HD)	(*Z*)-β-Farnesene (7.0%), germacrene D (24.5%), bicyclogermacrene (6.7%), β-bisabolene (8.1%), (*Z*)-α-bisabolene (39.3%)	---	[131]
*P. dilatatum* Rich.	Xambioá, Tocantins state, Brazil	Aerial parts (HD)	Germacrene D (8.5%), bicyclogermacrene (34.7%), spathulenol (35.2%)	---	[131]
*P. dilatatum* Rich.	Xambioá, Tocantins state, Brazil	Aerial parts (HD)	Germacrene D (15.2%), curzerene (28.7%), β-bisabolene (5.5%), (*Z*)-α-bisabolene (23.2%)	---	[131]
*P. dilatatum* Rich.	Carolina, Maranhão state, Brazil	Aerial parts (HD)	Limonene (19.4%), germacrene D (43.0%), bicyclogermacrene (13.2%)	---	[131]
*P. diospyrifolium* Kunth	Universidade de São Paulo, Brazil	Leaf (HD)	(*E*)-Nerolidol (18.2%), spathulenol (25.4%), caryophyllene oxide (7.7%), globulol (6.6%), humulene epoxide II (6.9%)	---	[35,60]
*P. diospyrifolium* Kunth	Universidade de São Paulo, Brazil	Floral (HD) ^a^	α-Copaene (47.7%), β-caryophyllene (12.3%), α-humulene (5.7%)	---	[60]
*P. diospyrifolium* Kunth	Maringá, Parana state, Brazil	Leaf (HD)	Limonene (8.5%), (*E*)-β-ocimene (5.8%), β-caryophyllene (16.8%), γ-muurolene (10.6%), *cis*-eudesma-6,11-diene (21.1%), germacrene B (6.2%)	Antifungal, agar diffusion assay (*Candida albicans*, *Candida parapsilosis*, *Candida tropicalis*)	[55]
*P. diospyrifolium* Kunth	Antonina, Paraná state, Brazil	Leaf (HD)	α-Pinene (6.7%), limonene (6.7%), α-copaene (5.4%), β-caryophyllene (7.4%), γ-gurjunene (6.9%), germacrene B (6.7%), selin-11-en-4α-ol (17.7%)	Antileishmanial (*L. amazonensis* promastigotes, IC_50_ 13.5 μg/mL; *L. amazonensis* axenic amastigotes, IC_50_ 76.1 μg/mL; anti-*Mycobacterium tuberculosis*, MIC 125 μg/mL)	[70]
*P. divaricatum* G. Mey.	Guaramiranga Mountain, Ceará state, Brazil	Leaf (HD)	α-Pinene (9.0–18.8%), β-pinene (19.9–25.3%), 1,8-cineole (8.9–9.6%), linalool (23.4–29.7%), germacrene D (6.3–6.5%)	---	[132]
*P. divaricatum* G. Mey.	Guaramiranga Mountain, Ceará state, Brazil	Floral (HD) ^a^	α-Pinene (6.3–17.6%), β-pinene (12.0–18.0%), α-phellandrene (4.6–10.0%), 1,8-cineole (0.7–12.0%), linalool (3.3–8.3%), β-caryophyllene (9.0–11.4%), germacrene D (0.9–6.1%), bicyclogermacrene (5.3–6.9%)	---	[132]
*P. divaricatum* G. Mey.	Marajó Island, Breves, Pará state, Brazil	Aerial parts (HD)	Eugenol (23.6%), methyleugenol (63.8%)	Antifungal (*Cladosporium cladosporioides*, MIC 0.5 μg/mL; *Cladosporium sphaerospermum*, 5.0 μg/mL); antioxidant (DPPH radical scavenging assay, IC_50_ 16.2 μg/mL)	[40]
*P. divaricatum* G. Mey.	Breves, Pará state, Brazil	Aerial parts (HD)	Eugenol (16.2%), methyleugenol (69.2%)	Insecticidal (*Solenopsis saevissima*, IC_50_ 453 μg/mL)	[41]
*P. divaricatum* G. Mey.	Itabuna, Bahia state, Brazil	Leaf (HD)	Safrole (98.0%)	Antibacterial (*Listeria monocytogenes*, MIC 156 μg/mL)	[44]
*P. divaricatum* G. Mey.	Rovira, Tolima, Colombia	Aerial parts (MWHD)	α-Pinene (11.4%), β-pinene (5.1%), α-phellandrene (6.1%), 1,8-cineole (18.3%), linalool (15.0%), β-caryophyllene (8.2%)	Antiprotozoal (*Trypanosoma cruzi* epimastigotes, IC_50_ 13.1 μg/mL; *Leishmania infantum* promastigotes, IC_50_ 73.3 μg/mL); cytotoxic (Vero cells, IC_50_ 89.8 μg/mL) Antifungal, broth dilution assay (*Trichophyton rubrum*, MIC 397 μg/mL; *Trichophyton mentagrophytes*, MIC 500 μg/mL); cytotoxic (Vero cells, IC_50_ 200 μg/mL)	[118,121]
*P. divaricatum* G. Mey.	Marajó Island, Breves, Pará state, Brazil	Aerial parts (HD)	Eugenol (7.9%), methyleugenol (77.1%)	Antifungal (*Fusarium solani* f. sp. *piperis*, IC_50_ 698 μg/mL)	[42]
*P. divaricatum* G. Mey.	Breves, Pará state, Brazil	Leaf (HD)	Methyleugenol (80.6–93.3%)	---	[43]
*P. dolichotrichum* Yunck.	Quibdó, Chocó, Colombia	Aerial parts (MWHD)	β-Elemene (6.4%), β-caryophyllene (9.8%), germacrene D (16.7%), bicyclogermacrene (6.2%), α-guaiene (5.9%)	---	[120]
*P. dotanum* Trel.	Monteverde, Costa Rica	Leaf (HD)	α-Thujene (5.2%), sabinene (50.4%), germacrene D (15.3%), bicyclogermacrene (7.4%)	---	[34]
*P. duckei* C. DC.	Adolpho Ducke Reserve, Manaus, Amazonas state, Brazil	Leaf (HD)	1,8-Cineole (5.8%), β-caryophyllene (27.1%), germacrene D (14.7%), bicyclogermacrene (5.2%), γ-eudesmol (17.9%)	Antileishmanial (*L. amazonensis* promastigotes, IC_50_ 46.0 μg/mL; *L. amazonensis* amastigotes, IC_50_ 42.4 μg/mL; *L. guyanensis* promastigotes, IC_50_ 15.2 μg/mL)	[72]
*P. dumosum* Rudge	Porto Velho, Rondônia state, Brazil	Leaf (HD)	α-Pinene (12.1%), β-pinene (16.0%), α-phellandrene (5.2%), β-caryophyllene (15.9%), aromadendrene (6.9%), bicyclogermacrene (16.2%)	---	[105]
*P. eriopodon* (Miq.) C. DC.	Pueblo Bello, Cesar, Colombia	Aerial parts (MWHD)	β-Caryophyllene (8.1%), β-selinene (5.0%), dillapiole (38.8%)	Antifungal, broth dilution assay (*Trichophyton mentagrophytes*, MIC 500 μg/mL); cytotoxic (Vero cells, IC_50_ 15.8 μg/mL)	[121]
*P. fimbriulatum* C. DC.	Altos de Campana National Park, Panama	Leaf (HD)	Linalool (5.3%), linalyl acetate (5.3%), β-caryophyllene (11.3%), germacrene D (12.8%)	---	[111]
*P. fimbriulatum* C. DC.	Monteverde, Costa Rica	Leaf (HD)	α-Pinene (10.2%), δ-elemene (9.4%), germacrene D (32.9%), bicyclogermacrene (8.1%)	Antibacterial (*Bacillus cereus*, MIC 39 μg/mL)	[34]
*P. friedrichsthalii* C. DC.	Pacayas, Cartago, Costa Rica	Leaf (HD)	α-Pinene (14.7%), camphene (5.2%), germacrene D (7.1%)	---	[133]
*P. friedrichsthalii* C. DC.	Pacayas, Cartago, Costa Rica	Floral (HD) ^a^	α-Pinene (13.4%), β-phellandrene (5.2%), *trans-p*-menth-2-en-1-ol (7.0%), *cis-p*-menth-2-en-1-ol (5.1%)	---	[133]
*P. friedrichsthalii* C. DC.	Fortuma, Quebrada Honda, Chiriqui, Panama	Leaf (HD)	Germacrene D (9.6%), α-selinene (12.0%), β-selinene (7.9%), selin-11-en-4α-ol (12.8%)	---	[133]
*P. gaudichaudianum* Kunth	Sapiranga, Rio Grande do Sul state, Brazil	Leaf (HD)	β-Pinene (5.6%), β-caryophyllene (17.4%), α-humulene (37.5%), *allo*-aromadendrene (7.7%)	---	[134]
*P. gaudichaudianum* Kunth	Universidade de São Paulo, Brazil	Aerial parts (HD)	β-Caryophyllene (12.1%), α-humulene (13.3%), β-selinene (15.7%), α-selinene (16.6%)	---	[135]
*P. gaudichaudianum* Kunth	Universidade de São Paulo, Brazil	Aerial parts (HD)	β-Caryophyllene (19.3%), α-humulene (29.2%), α-selinene (8.9%)	---	[135]
*P. gaudichaudianum* Kunth	State of Rondônia, Brazil	Leaf (HD)	Aromadendrene (15.6%), ishwarane (10.0%), β-selinene (10.5%), viridiflorol (27.5%), selin-11-en-4α-ol (8.5%)	Mosquito larvicidal (*Aedes aegypti*, LC_50_ 121 μg/mL)	[136]
*P. gaudichaudianum* Kunth	Riozinho, Rio Grande do Sul state, Brazil	Leaf (HD)	β-Caryophyllene (8.9%), α-humulene (16.5%), bicyclogermacrene (7.4%), (*E*)-nerolidol (22.4%)	Cytotoxic (V79 Chinese hamster lung cells, IC_50_ 4.0 μg/mL)	[137]
*P. gaudichaudianum* Kunth	Universidade de São Paulo, Brazil	Leaf (HD)	β-Caryophyllene (15.6%), α-humulene (23.4%), β-selinene (6.6%), viridiflorene (8.1%), hinesol (6.4%), α-cadinol (7.0%)	Antifungal, broth dilution assay (*Candida krusei*, MIC 31.25 μg/mL)	[35]
*P. gaudichaudianum* Kunth	Riozinho, Rio Grande do Sul state, Brazil	Leaf (HD)	β-Caryophyllene (7.5%), α-humulene (21.3%), bicyclogermacrene (13.2%), (*E*)-nerolidol (22.1%)	Not mutagenic (*Saccharomyces cerevisiae*); EO and nerolidol generate reactive oxygen species	[138]
*P. gaudichaudianum* Kunth	Pariquera-Açu, São Paulo state, Brazil	Leaf (HD)	α-Pinene (12.2%), β-pinene (7.0%), β-caryophyllene (8.5%), *trans*-β-guaiene (6.9%), (*E*)-nerolidol (17.5%), caryophyllene oxide (8.5%)	---	[31]
*P. gaudichaudianum* Kunth	Antonina, Paraná state, Brazil	Leaf (HD)	δ-3-Carene (5.9%), γ-elemene (5.4%), δ-cadinene (45.3%)	Antileishmanial (*L. amazonensis* promastigotes, IC_50_ 93.5 μg/mL)	[70]
*P. glabratum* Kunth	Reserva da Matinha, Ilhéus, Bahia state, Brazil	Leaf (HD)	(*Z*)-Caryophyllene (5.2%), β-caryophyllene (14.6%), δ-cadinene (6.3%), (*E*)-nerolidol (5.3%), longiborneol (12.0%)	---	[125]
*P. glabrescens* (Miq.) C. DC.	Monteverde, Costa Rica	Leaf (HD)	α-Pinene (26.0%), limonene (56.6%)	Cytotoxic (MCF-7 human breast adenocarcinoma)	[34]
*P. grande* Vahl	Parque Nacional Camino de Cruces, Panama	Leaf (HD)	α-Pinene (6.3%), β-pinene (14.5%), γ-terpinene (8.0%), *p*-cymene (43.9%)	---	[47]
*P. heterophyllum* Ruiz & Pav.	Estancia, Bolivia	Leaf (SD)	α-Pinene (9.3%), β-pinene (6.2%), 1,8-cineole (39.0%), (*E*)-β-ocimene (6.5%), asaricin (8.8%)	---	[101]
*P. hispidinervum* C. DC.	Porto Alegre, Rio Grande do Sul state, Brazil	Leaf (HD)	Terpinolene (5.4%), safrole (85.1%)	Amebicidal (*Acanthamoeba polyphaga* trophozoites, LC_50_ 66 μg/mL)	[139]
*P. hispidum* Sw.	Rondônia state, Brazil	Leaf (SD)	α-Pinene (5.2%), camphene (15.6%), β-phellandrene (9.7%), β-caryophyllene (5.4%), α-guaiene (11.5%), γ-cadinene (25.1%), γ-elemene (10.9%) ^c^	---	[112]
*P. hispidum* Sw.	Pinar del Río, Cuba	Leaf (HD)	Curzerene (12.9%), elemol (7.6%), γ-eudesmol (9.3%), β-eudesmol (17.5%), α-eudesmol (8.1%), 14-Hydroxy-α-muurolene (5.0%)	---	[46]
*P. hispidum* Sw.	Fazenda Sucupira, Embrapa, Brasília, Brazil	Leaf (HD)	α-Pinene (9.0%), β-pinene (19.7%), δ-3-carene (7.4%), spathulenol (6.2%), α-cadinol (6.9%)	---	[33]
*P. hispidum* Sw.	Pacurita, Chocó, Colombia	Leaf (HD)	β-Elemene (5.1%), β-caryophyllene (5.1%), (*E*)-nerolidol (23.6%), caryophyllene oxide (5.4%)	---	[48]
*P. hispidum* Sw.	Fênix, Paraná state, Brazil	Floral (HD) ^a^	α-Pinene (7.1–13.9%), β-pinene (7.5–13.3%), α-copaene (28.7–36.2%)	---	[108]
*P. hispidum* Sw.	Chiguará, Mérida state, Venezuela	Leaf (HD)	α-Pinene (15.3%), β-pinene (14.8%), δ-3-carene (6.9%), β-elemene (8.1%), β-caryophyllene (6.2%), germacrene B (5.2%), spathulenol (5.0%), caryophyllene oxide (7.8%)	Antibacterial (*Bacillus subtilis*, MIC 12.5 μg/mL; *Bacillus cereus*, MIC 12.5 μg/mL; *Staphylococcus aureus*, MIC 12.5 μg/mL; *Staphylococcus epidermidis*, MIC 12.5 μg/mL; *Staphylococcus saprophyticus*, MIC 12.5 μg/mL; *Enterococcus faecalis*, MIC 15.0 μg/mL), antifungal (*Candida albicans*, MIC 200 μg/mL), cytotoxic (HeLa human cervical carcinoma, IC_50_ 36.6 μg/mL; A-549 human lung carcinoma, IC_50_ 37.5 μg/mL; MCF-7 human breast adenocarcinoma, IC_50_ 34.2 μg/mL)	[97]
*P. hispidum* Sw.	Reserva da Matinha, Ilhéus, Bahia state, Brazil	Leaf (HD)	α-Pinene (6.6%), β-pinene (12.0%), khusimene (12.1%), γ-cadinene (13.2%), δ-cadinene (6.3%), ledol (8.8%)	---	[125]
*P. hispidum* Sw.	Carajás National Forest, Parauapebas, Pará state, Brazil	Aerial parts (HD)	δ-3-Carene (9.1%), limonene (6.9%), α-copaene (7.3%), β-caryophyllene (10.5%), α-humulene (9.5%), β-selinene (5.1%), caryophyllene oxide (5.9%)	Antifungal, TLC bioautography (*Cladosporium cladosporioides*, *Cladosporium sphaerospermum*); enzyme inhibitory, TLC bioautography (acetylcholinesterase)	[61]
*P. hispidum* Sw.	Atrato, Chocó, Colombia	Aerial parts (MWHD)	β-Elemene (5.1%), β-caryophyllene (5.1%), (*E*)-nerolidol (23.6%), caryophyllene oxide (5.4%)	Antifungal, broth dilution assay (*Fusarium oxysporum*, MIC 500 μg/mL; *Trichophyton rubrum*, MIC 99 μg/mL; *Trichophyton mentagrophytes*, MIC 125 μg/mL); cytotoxic (Vero cells, IC_50_ 51.7 μg/mL)	[121]
*P. hispidum* Sw.	Altos de Campana, Panama	Leaf (HD)	Piperitone (10.0%), dillapiole (57.7%)	Mosquito larvicidal (*Aedes aegypti*, LC_100_ 250 μg/mL)	[47]
*P. hostmannianum* (Miq.) C. DC.	State of Rondônia, Brazil	Leaf (HD)	Piperitone (5.6%), germacrene D (6.8%), asaricin (27.4%), myristicin (20.3%), dillapiole (7.7%)	Mosquito larvicidal (*Aedes aegypti*, LC_50_ 54 μg/mL)	[136]
*P. humaytanum* Yunck.	State of Rondônia, Brazil	Leaf (HD)	β-Selinene (15.8%), sesquicineole (5.0%), spathulenol (6.3%), caryophyllene oxide (16.6%), β-oplopenone (6.0%)	Mosquito larvicidal (*Aedes aegypti*, LC_50_ 156 μg/mL)	[136]
*P. ilheusense* Yunck.	Ilheus, Bahia, Brazil	Leaf (HD)	β-Caryophyllene (11.8%), γ-cadinene (6.9%), germacrene B (7.2%), gleenol (7.5%), patchouli alcohol (11.1%)	Antimicrobial, agar diffusion assay (*Bacillus subtilis*, *Staphylococcus aureus*, *Candida albicans*, *Candida crusei*, *Candida parapsilosis*)	[57]
*P. imperiale* (Miq.) C. DC.	Monteverde, Costa Rica	Leaf (HD)	β-Elemene (5.2%), β-caryophyllene (25.5%), α-guaiene (7.6%), germacrene D (5.5%), bicyclogermacrene (19.7%), germacrene A (8.5%), α-bulnesene (10.8%), dillapiole (6.7%)	Antibacterial (*Bacillus cereus*, MIC 156 μg/mL), cytotoxic (MCF-7 human breast adenocarcinoma)	[34]
*P. jacquemontianum* Kunth	Lachuá, Alta Verapaz, Guatemala	Leaf (HD)	Linalool (69.4%), (*E*)-nerolidol (8.0%)	---	[140]
*P. jacquemontianum* Kunth	Parque Nacional Soberania, Panama	Leaf (HD)	α-Pinene (9.6%), β-pinene (10.1%), α-phellandrene (13.8%), limonene (12.2%), *p*-cymene (7.4%), linalool (14.5%)	---	[47]
*P. klotzschianum* (Kunth) C. DC.	Vila do Riacho, Gimuna Forest, Aracruz, Espírito Santo, Brazil	Leaf (HD)	4-Butyl-1,2-methylenedioxybenzene (81.0%), γ-asarone (9.1%)	---	[141]
*P. klotzschianum* (Kunth) C. DC.	Vila do Riacho, Gimuna Forest, Aracruz, Espírito Santo, Brazil	Root (HD)	4-Butyl-1,2-methylenedioxybenzene (96.2%)	Mosquito larvicidal activity (*Aedes aegypti*, LC_50_ 10.0 μg/mL)	[141]
*P. klotzschianum* (Kunth) C. DC.	Vila do Riacho, Gimuna Forest, Aracruz, Espírito Santo, Brazil	Seed (HD)	α-Phellandrene (17.0%), *p*-cymene (7.4%), limonene (17.8%), 4-Butyl-1,2-methylenedioxybenzene (36.9%), α-*trans*-bergamotene (8.8%)	Mosquito larvicidal activity (*Aedes aegypti*, LC_50_ 13.3 μg/mL)	[141]
*P. klotzschianum* (Kunth) C. DC.	Vila do Riacho, Gimuna Forest, Aracruz, Espírito Santo, Brazil	Stem (HD)	4-Butyl-1,2-methylenedioxybenzene (84.8%), γ-asarone (5.4%)	---	[141]
*P. krukoffii* Yunck.	Carajás National Forest, Parauapebas, Pará state, Brazil	Aerial parts (HD)	β-Elemene (1.7–8.2%), myristicin (26.7–40.6%), τ-muurolol (0.2–5.7%), apiole (25.3–34.1%)	---	[142]
*P. lanceifolium* Kunth	San Isidro del Tejar, Costa Rica	Leaf (HD)	β-Caryophyllene (20.6%), germacrene D (12.5%), elemicin (24.4%), apiole (11.7%)	---	[143]
*P. lanceifolium* Kunth	San Isidro del Tejar, Costa Rica	Floral (HD) ^a^	α-Pinene (13.7%), β-pinene (15.8%), γ-terpinene (6.9%), β-caryophyllene (5.1%), elemicin (16.4%), apiole (9.8%)	---	[143]
*P. lanceifolium* Kunth	Monteverde, Costa Rica	Leaf (HD)	Dillapiole (74.6%)	---	[34]
*P. lanceifolium* Kunth	Bagadó, Chocó, Colombia	Aerial parts (MWHD)	β-Pinene (5.4%), β-caryophyllene (11.6%), germacrene D (10.7%), β-selinene (7.8%), δ-cadinene (6.1%), caryophyllene oxide (5.9%)	Antiprotozoal (*Trypanosoma cruzi* epimastigotes, IC_50_ 7.48 μg/mL; *Leishmania infantum* promastigotes, IC_50_ 37.8 μg/mL); cytotoxic (Vero cells, IC_50_ 46.0 μg/mL; THP-1 human monocytic leukemia, IC_50_ 55.7 μg/mL)	[118]
*P. leptorum* Kunth	Monte Alegre do Sul, São Paulo state, Brazil	Leaf (HD)	Seychellene (34.7%), caryophyllene oxide (12.5%)	---	[31]
*P. longispicum* C. DC.	Altos de Campana, Panama	Leaf (HD)	β-Caryophyllene (45.2%), caryophyllene oxide (5.5%)	Mosquito larvicidal (*Aedes aegypti*, LC_100_ 250 μg/mL)	[47]
*P. lucaeanum* var. *grandifolium* Yunck.	Rio de Janeiro state, Brazil	Leaf (HD)	α-Pinene (30.0%), β-caryophyllene (5.0%), α-zingiberene (30.4%), β-bisabolene (8.9%), β-sesquiphellandrene (11.1%)	Antiparasitic (*Plasmodium falciparum* W2, IC_50_ 2.65 μg/mL)	[73]
*P. madeiranum* Yunck.	Reserva da Matinha, Ilhéus, Bahia state, Brazil	Leaf (HD)	β-Caryophyllene (11.2%), germacrene D-4-ol (11.1%), 1,10-di-*epi*-cubenol (7.0%), α-bisabolol (7.1%), *epi*-α-bisabolol (5.4%)	---	[125]
*P. malacophyllum* (C. Presl) C. DC.	Florianópolis, Santa Catarina, Brazil	Leaf (HD)	α-Pinene (5.0%), camphene (30.8%), camphor (32.8%)	Antibacterial (*Staphylococcus aureus*, MIC 3700 μg/mL; *Bacillus cereus*, MIC 1850 μg/mL; *Acinetobacter baumanii*, MIC 3700 μg/mL; *Escherichia coli*, MIC 1850 μg/mL; *Pseudomonas aeruginosa*, MIC 3700 μg/mL); antifungal (*Epidermophyton flocosum*, MIC 1000 μg/mL; *Microsporum gypseum*, MIC 1000 μg/mL; *Trichophyton mentagrophytes*, MIC 500 μg/mL; *Trichophyton rubrum*, MIC 1000 μg/mL; *Candida albicans*, MIC 1000 μg/mL; *Cryptococcus neoformans*, MIC 500 μg/mL); antiparasitic (*Trypanosoma cruzi* epimastigotes, IC_50_ 312 μg/mL)	[56]
*P. manausense* Yunck.	Ananindeua, Pará state, Brazil	Aerial parts (HD)	α-Pinene (5.2–6.6%), β-pinene (4.7–6.5%), β-caryophyllene (7.7–8.5%), germacrene D (3.5–6.1%), bicyclogermacrene (32.0–34.0%), δ-cadinene (5.8–7.0%), gleenol (6.8–9.4%)	---	[144]
*P. manausense* Yunck.	Acará, Pará state, Brazil	Aerial parts (HD)	α-Pinene (9.1%), β-pinene (9.2%), β-caryophyllene (5.9%), bicyclogermacrene (41.0%), δ-cadinene (5.8%)	---	[144]
*P. manausense* Yunck.	Marituba, Pará state, Brazil	Aerial parts (HD)	β-Caryophyllene (6.0%), aromadendrene (5.0%), bicyclogermacrene (7.8%), spathulenol (15.0%), globulol (9.4%), α-muurolol (7.6%)	---	[144]
*P. marginatum* Jacq.	Itacoatiara, Amazonas State, Brazil	Leaf (HD)	(*E*)-β-Ocimene (5.2%), α-copaene (5.6%), β-caryophyllene (9.1%), γ-elemene (8.5%), propiopiperone (18.2%)	---	[145]
*P. marginatum* Jacq.	Itacoatiara, Amazonas State, Brazil	Stem (HD)	δ-3-Carene (6.9%), β-caryophyllene (11.6%), myristicin (19.3%), propiopiperone (18.6%)	---	[145]
*P. marginatum* Jacq.	Monteverde, Costa Rica	Aerial parts (HD)	*p*-Cymene (7.1%), estragole (6.6%), *p*-anisaldehyde (22.0%), (*E*)-anethole (45.9%), anisyl methyl ketone (14.2%)	---	[49]
*P. marginatum* Jacq.	Cultivated (State University of Campinas, São Paulo, Brazil)	Leaf (HD)	---	Antibacterial (*Escherichia coli*, MIC 700 μg/mL)	[52]
*P. marginatum* Jacq.	Monte Alegre, Pará state, Brazil	Leaf (HD)	(*E*)-β-Ocimene (5.6%), safrole (63.9%), methyleugenol (5.9%), propiopiperone (7.3%)	---	[45]
*P. marginatum* Jacq.	Xambioá, Tocantins state, Brazil	Leaf (HD)	Safrole (52.3–52.5%), myristicin (6.3–9.3%), propiopiperone (11.8–14.1%)	---	[45]
*P. marginatum* Jacq.	Nazaré, Tocantins state, Brazil	Leaf (HD)	Safrole (41.1%), myristicin (8.2%), propiopiperone (30.4%)	---	[45]
*P. marginatum* Jacq.	Monte Alegre, Pará state, Brazil	Leaf (HD)	(*Z*)-β-Ocimene (5.3%), (*E*)-β-ocimene (13.5%), safrole (23.9%), β-caryophyllene (6.0%), propiopiperone (33.2%)	---	[45]
*P. marginatum* Jacq.	Belém, Pará state, Brazil	Leaf (HD)	*p*-Mentha-1(7),8-diene (39.0%), (*E*)-β-ocimene (9.8%), propiopiperone (19.0%)	---	[45]
*P. marginatum* Jacq.	Alter do Chão, Pará state, Brazil	Leaf (HD)	α-Pinene (5.0%), *p*-mentha-1(7),8-diene (34.8%), (*E*)-β-ocimene (8.7%), propiopiperone (23.1%), elemicin (6.5%)	---	[45]
*P. marginatum* Jacq.	Belterra, Pará state, Brazil	Leaf (HD)	*p*-Mentha-1(7),8-diene (22.9%), (*E*)-β-ocimene (8.2%), propiopiperone (40.7%)	---	[45]
*P. marginatum* Jacq.	Melgaço, Pará state, Brazil	Leaf (HD)	(*E*)-β-Ocimene (8.0%), safrole (10.4%), germacrene D (8.1%), bicyclogermacrene (6.4%), myristicin (16.0%), propiopiperone (17.4%)	---	[45]
*P. marginatum* Jacq.	Xinguara, Pará state, Brazil	Leaf (HD)	(*Z*)-β-Ocimene (8.6%), (*E*)-β-ocimene (15.2%), germacrene D (10.4%), myristicin (5.4%), propiopiperone (14.5%), τ-muurolol (5.0%)	---	[45]
*P. marginatum* Jacq.	Manaus, Amazonas state, Brazil	Leaf (HD)	Safrole (6.4%), α-copaene (7.4%), β-caryophyllene (9.5%), germacrene D (5.5%), propiopiperone (25.0%)	---	[45]
*P. marginatum* Jacq.	Macapá, Amapá state, Brazil	Leaf (HD)	(*E*)-β-Ocimene (5.5%), β-caryophyllene (10.6%), myristicin (9.6%), propiopiperone (22.9%)	---	[45]
*P. marginatum* Jacq.	Monte Alegre, Pará state, Brazil	Leaf (HD)	(*Z*)-β-Ocimene (5.7%), (*E*)-β-ocimene (13.5%), β-caryophyllene (9.3%), propiopiperone (40.2%)	---	[45]
*P. marginatum* Jacq.	Viseu, Pará state, Brazil	Leaf (HD)	γ-Terpinene (14.4%), myristicin (5.0%), propiopiperone (29.6%), spathulenol (6.6%)	---	[45]
*P. marginatum* Jacq.	Alta Floresta, Mato Grosso state, Brazil	Leaf (HD)	γ-Terpinene (8.6%), myristicin (5.5%), propiopiperone (18.4%)	---	[45]
*P. marginatum* Jacq.	Manaus, Amazonas state, Brazil	Leaf (HD)	γ-Terpinene (6.5%), safrole (5.7%), β-caryophyllene (13.3%), germacrene D (8.7%), propiopiperone (7.9%)	---	[45]
*P. marginatum* Jacq.	Manaus, Amazonas state, Brazil	Leaf (HD)	(*Z*)-β-Ocimene (5.2%), (*E*)-β-ocimene (8.7%), α-copaene (11.4%), β-caryophyllene (10.2%), germacrene D (7.6%), bicyclogermacrene (8.2%), propiopiperone (10.4%)	---	[45]
*P. marginatum* Jacq.	Salvaterra, Pará state, Brazil	Leaf (HD)	*p*-Mentha-1(7),8-diene (5.2%), (*Z*)-anethole (8.4%), (*E*)-anethole (16.5%), isoosmorhizole (17.4%), (*E*)-isoosmorhizole (29.1%)	---	[45]
*P. marginatum* Jacq.	Manaus, Amazonas state, Brazil	Leaf (HD)	(*Z*)-Anethole (6.0%), (*E*)-anethole (26.4%), isoosmorhizole (11.2%), (*E*)-isoosmorhizole (32.2%)	---	[45]
*P. marginatum* Jacq.	Óbidos, Pará state, Brazil	Leaf (HD)	(*E*)-Anethole (13.6%), isoosmorhizole (24.5%), (*E*)-isoosmorhizole (46.8%)	---	[45]
*P. marginatum* Jacq.	Medicilândia, Pará state, Brazil	Leaf (HD)	β-Caryophyllene (6.7%), (*E*)-isoosmorhizole (15.8%), crocatone (21.9%), 2′-methoxy-4′,5′-methylenedioxypropiophenone (26.3%)	---	[45]
*P. marginatum* Jacq.	Paredão, Roraima state, Brazil	Leaf (HD)	β-Caryophyllene (13.6%), bicyclogermacrene (11.7%), (*Z*)-asarone (8.8%), exalatacin (7.9%), (*E*)-asarone (10.8%)	---	[45]
*P. marginatum* Jacq.	Venadillo, Tolima, Colombia	Aerial parts (HD)	α-Phellandrene (11.1%), limonene (7.5%), β-caryophyllene (11.0%), elemicin (18.0%), isoelemicin (9.2%)	---	[120]
*P. marginatum* Jacq.	Universidade Federal Rural de Pernambuco, Recife, Brazil	Leaf (HD)	β-Caryophyllene (7.5%), α-acoradiene (5.1%), bicyclogermacrene (9.4%), elemol (9.7%), (*Z*)-asarone (30.4%), patchouli alcohol (16.0%), (*E*)-asarone (6.4%)	Mosquito larvicidal (*Aedes aegypti*, LC_50_ 23.8 μg/mL)	[146]
*P. marginatum* Jacq.	Universidade Federal Rural de Pernambuco, Recife, Brazil	Floral (HD) ^a^	α-Copaene (9.4%), β-caryophyllene (13.1%), α-acoradiene (9.7%), patchouli alcohol (23.4%), (*E*)-asarone (22.1%)	Mosquito larvicidal (*Aedes aegypti*, LC_50_ 19.9 μg/mL)	[146]
*P. marginatum* Jacq.	Universidade Federal Rural de Pernambuco, Recife, Brazil	Stem (HD)	β-Caryophyllene (6.8%), seychellene (5.8%), elemicin (6.9%), (*Z*)-asarone (8.5%), patchouli alcohol (25.7%), (*E*)-asarone (32.6%)	Mosquito larvicidal (*Aedes aegypti*, LC_50_ 19.9 μg/mL)	[146]
*P. marginatum* Jacq.	Belém, Pará state, Brazil	Aerial parts (HD)	*p*-Mentha-1(7),8-diene (39.0%), (*E*)-β-ocimene (9.8%), propiopiperone (19.0%)	Insecticidal (*Solenopsis saevissima*, IC_50_ 240 μg/mL)	[41]
*P. marginatum* Jacq.	Manaus, Amazonas state, Brazil	Aerial parts (HD)	(*E*)-Anethole (26.4%), isoosmorhizole (11.2%), (*E*)-isoosmorhizole (32.2%)	Insecticidal (*Solenopsis saevissima*, IC_50_ 439 μg/mL)	[41]
*P. marginatum* Jacq.	Venadillo, Tolima, Colombia	Aerial parts (MWHD)	α-Phellandrene (11.2%), limonene (7.6%), β-caryophyllene (11.1%), elemicin (18.4%), isoelemicin (9.3%)	Antiprotozoal (*Trypanosoma cruzi* epimastigotes, IC_50_ 16.2 μg/mL; *Leishmania infantum* promastigotes, IC_50_ 88.7 μg/mL); cytotoxic (Vero cells, IC_50_ 40.2 μg/mL) Antifungal, broth dilution assay (*Trichophyton rubrum*, MIC 500 μg/mL; *Trichophyton mentagrophytes*, MIC 250 μg/mL)	[118,121]
*P. marginatum* Jacq.	Belém, Pará state, Brazil	Aerial parts (HD)	β-Caryophyllene (5.0%), propiopiperone (21.8%), elemol (5.9%)	Antifungal, TLC bioautography (*Cladosporium cladosporioides*, *Cladosporium sphareospermum*), enzyme inhibitory (acetylcholinesterase)	[62]
*P. mikanianum* (Kunth) Steud.	Sapiranga, Rio Grande do Sul state, Brazil	Leaf (HD)	α-Pinene (6.5%), myrcene (5.6%), limonene (14.8%), β-caryophyllene (10.5%), bicyclogermacrene (14.3%)	---	[134]
*P. mikanianum* (Kunth) Steud.	Atalanta, Santa Catarina state, Brazil	Leaf (HD)	Safrole (82.0%)	---	[147]
*P. mikanianum* (Kunth) Steud.	Curitiba, Paraná state, Brazil	Leaf (HD)	Bicyclogermacrene (5.3%), (*Z*)-isoelemicin (21.5%), (*E*)-asarone (11.6%), β-vetivone (33.5%)	---	[148]
*P. mikanianum* (Kunth) Steud.	Picada Café, Rio Grando do Sul state, Brazil	Aerial parts (HD)	Bicyclogermacrene (6.6%), germacrene B (7.8%), α-cadinol (5.1%), apiole (64.9%)	Acaricidal (*Rhipicephalus* (*Boophilus*) *microplus*, LC_50_ 2.33 μL/mL)	[106]
*P. mikanianum* (Kunth) Steud.	Atalanta, Santa Catarina state, Brazil	Leaf (HD)	α-Thujene (6.0%), safrole (72.4%)	---	[70]
*P. mollicomum* Kunth	Cultivated (State University of Campinas, São Paulo, Brazil)	Leaf (HD)	---	Antibacterial (*Escherichia coli*, MIC 1000 μg/mL)	[52]
*P. mollicomum* Kunth	Cultivated, FIOCRUZ, Rio de Janeiro, Brazil	Leaf (HD)	(*Z*)-β-Ocimene (14.1%), (*E*)-β-ocimene (12.1%), germacrene D (10.8%), germacrene B (13.4%), myrtenic acid (7.5%), α-bisabolol (9.9%), (*E*)-nerolidol (9.6%)	Antinociceptive (mouse model, 1 mg/kg)	[91]
*P. mosenii* C. DC.	Antonina, Paraná state, Brazil	Leaf (HD)	β-Caryophyllene (8.6%), α-humulene (11.3%), bicyclogermacrene (7.4%), caryophyllene oxide (12.1%), viridiflorol (5.8%), humulene epoxide II (6.3%)	Antileishmanial (*L. amazonensis* promastigotes, IC_50_ 17.4 μg/mL; *L. amazonensis* axenic amastigotes, IC_50_ >200 μg/mL), anti-*Mycobacterium tuberculosis* (MIC 250 μg/mL)	[70]
*P. multiplinervium* C. DC.	Parque Nacional Soberania, Panama	Leaf (HD)	α-Pinene (7.1%), β-pinene (7.9%), α-phellandrene (11.8%), limonene (11.4%), *p*-cymene (9.0%), linalool (16.5%), (*E*)-nerolidol (5.5%)	---	[47]
*P. nemorense* C. DC.	Monteverde, Costa Rica	Leaf (HD)	α-Phellandrene (8.8%), limonene (6.3%), α-copaene (5.7%), β-bourbonene (14.0%), β-caryophyllene (5.6%), β-copaene (15.0%), γ-elemene (6.8%), germacrene D (8.4%), bicyclogermacrene (7.5%)	---	[34]
*P. oblanceolatum* Trel.	Monteverde, Costa Rica	Leaf (HD)	α-Pinene (6.2%), linalool (11.3%), β-caryophyllene (6.8%), germacrene D (8.9%), δ-amorphene (9.0%)	Antibacterial (*Bacillus cereus*, MIC 78 μg/mL), cytotoxic (MCF-7 human breast adenocarcinoma)	[34]
*P. obliquum* Ruiz & Pav.	Altos de Campana National Park, Panama	Leaf (HD)	β-Caryophyllene (27.6%), spathulenol (10.6%), caryophyllene oxide (8.3%)	---	[111]
*P. obliquum* Ruiz & Pav.	Wasak'entsa reserve, Ecuador	Aerial parts (HD)	γ-Terpinene (17.1%), terpinolene (11.5%), safrole (45.9%)	---	[65]
*P. obrutum* Trel. & Yunck.	Samurindó, Chocó, Colombia	Aerial parts (MWHD)	Linalool (15.8%), β-elemene (7.6%), α-humulene (6.4%), (*E*)-nerolidol (5.8%)	Antiprotozoal (*Trypanosoma cruzi* epimastigotes, IC_50_ 29.3 μg/mL; *Leishmania infantum* promastigotes, IC_50_ 35.9 μg/mL; *L. infantum* amastigotes, IC_50_ 89.0 μg/mL); cytotoxic (Vero cells, IC_50_ 45.3 μg/mL)	[118]
*P. ovatum* Vahl	Fazenda Sucupira, Embrapa, Brasília, Brazil	Leaf (HD)	α-Pinene (23.1%), β-pinene (14.2%), β-caryophyllene (5.3%), germacrene D (10.3%), *epi*-cubebol (10.7%)	---	[33]
*P. peltatum* L. [syn. *Pothomorphe peltata* (L.) Miq.]	Pinar del Río, Cuba	Leaf (HD)	α-Copaene (5.2%), *trans*-calamenene (5.4%), spathulenol (9.0%), caryophyllene oxide (22.9%)	---	[27]
*P. permucronatum* Yunck.	Tijuca Forest, Rio de Janeiro state, Brazil	Leaf (HD)	β-Caryophyllene (6.8%), δ-cadinene (12.7%), α-cadinol (6.9%)	---	[149]
*P. permucronatum* Yunck.	State of Rondônia, Brazil	Leaf (HD)	Asaricin (8.6%), myristicin (25.6%), elemicin (9.9%), dillapiole (54.7%)	Mosquito larvicidal (*Aedes aegypti*, LC_50_ 36 μg/mL)	[136]
*P. plurinervosum* Yunck.	Egler Reserva, Amazonas, Brazil	Aerial parts (HD)	1,8-Cineole (31.6%), β-caryophyllene (6.6%), (*E*)-nerolidol (6.4%), caryophyllene oxide (5.7%), guaiol (6.2%), α-cadinol (8.5%)	---	[129]
*P. pseudolindenii* C. DC.	Turrialba, Cartago, Costa Rica	Leaf (HD)	β-Pinene (6.7%), β-elemene (15.0%), β-caryophyllene (11.8%), α-humulene (7.0%), germacrene D (9.0%), germacrene B (5.4%)	---	[133]
*P. regnellii* (Miq.) C. DC.	Universidade de São Paulo, Brazil	Aerial parts (HD)	β-Caryophyllene (23.4%), (*E*)-nerolidol (13.7%), spathulenol (11.1%), globulol (6.1%)	---	[135]
*P. regnellii* (Miq.) C. DC.	Universidade de São Paulo, Brazil	Leaf (HD)	Myrcene (52.6%), linalool (15.9%), β-caryophyllene (8.5%)	Antimicrobial, agar diffusion assay (*Staphylococcus aureus*, *Candida albicans*)	[38]
*P. regnellii* (Miq.) C. DC.	Cultivated (State University of Campinas, São Paulo, Brazil)	Leaf (HD)	---	Antibacterial (*Escherichia coli*, MIC 300 μg/mL)	[52]
*P. regnellii* (Miq.) C. DC.	Universidade de São Paulo, Brazil	Leaf (HD)	β-Pinene (13.3%), myrcene (15.5%), β-caryophyllene (7.2%), aromadendrene (8.3%), bicyclogermacrene (9.7%), (*E*)-nerolidol (8.4%), spathulenol (7.8%)	---	[35]
*P. regnellii* (Miq) C. DC. var. *regnellii* (C. DC.) Yunck	Universidade de São Paulo, Brazil	Leaf (HD)	β-caryophyllene (8.2–9.5%), germacrene D (45.6–51.4%) and α-chamigrene (8.9–11.3%)	Cytotoxic (B16F10-Nex2 murine melanoma, IC_50_ 66 μg/mL; A2058 human melanoma, IC_50_ 57 μg/mL; HeLa human cervical carcinoma, IC_50_ 13 μg/mL; SiHa human cervical IC_50_ 71 μg/mL; HCT human colon carcinoma, IC_50_ 61 μg/mL; SKBR3 breast cancer, IC_50_ 79 μg/mL; U87 human glioblastoma, IC_50_ 71 μg/mL; β-caryophyllene, germacrene D, α-chamigrene cytotoxic to HeLa cells: IC_50_ 11, 7, 32 μg/mL, respectively)	[98]
*P. renitens* (Miq.) Yunck.	Mirante da Serra, Rondonia, Brazil	Aerial parts (HD)	α-Pinene (12.5%), camphene (5.6%), β-pinene (12.4%), (*Z*)-caryophyllene (6.9%), germacrene D (13.8%), bicyclogermacrene (6.6%), guaiol (13.9%), eudesm-7(11)-en-4-ol (9.3%)	---	[150]
*P. reticulatum* L.	Costa Arriba, Rio Cascajal, Colon, Panama	Leaf (HD)	β-Elemene (16.1%), β-selinene (19.0%), α-selinene (15.5%), spathulenol (6.1%)	---	[47]
*P. rivinoides* Kunth	Cultivated, FIOCRUZ, Rio de Janeiro, Brazil	Leaf (HD)	α-Pinene (32.9%), β-pinene (20.7%), β-caryophyllene (7.6%), germacrene B (6.7%)	Antinociceptive (mouse model, 1 mg/kg)	[91]
*P. rivinoides* Kunth	Ubatuba, São Paulo state, Brazil	Leaf (HD)	α-Pinene (73.2%), β-pinene (5.2%)	---	[31]
*P. rivinoides* Kunth	Antonina, Paraná state, Brazil	Leaf (HD)	β-Caryophyllene (6.6%), α-humulene (10.0%), dehydroaromadendrane (7.8%), bicyclogermacrene (11.8%), (*Z*)-α-bisabolene (10.9%), spathulenol (5.1%)	Antileishmanial (*L. amazonensis* promastigotes, IC_50_ 10.9 μg/mL; *L. amazonensis* axenic amastigotes, IC_50_ > 200 μg/mL), anti-*Mycobacterium tuberculosis* (MIC 125 μg/mL)	[70]
*P. septuplinervium* (Miq.) C. DC.	Pandó, Chocó, Colombia	Aerial parts (MWHD)	β-Caryophyllene (5.0%), *epi*-cubebol (9.0%), δ-cadinene (10.9%), germacrene D-4-ol (5.6%), viridiflorol (7.9%)	Antiprotozoal (*Trypanosoma cruzi* epimastigotes, IC_50_ 14.0 μg/mL; *Leishmania infantum* promastigotes, IC_50_ 30.1 μg/mL; *L. infantum* amastigotes, IC_50_ 64.8 μg/mL); cytotoxic (Vero cells, IC_50_ 42.7 μg/mL; THP-1 human monocytic leukemia, IC_50_ 48.8 μg/mL)	[118]
*P. solmsianum* C. DC.	Teresópolis, Rio de Janeiro state, Brazil	Leaf (HD)	δ-3-Carene (23.3%), asaricin (39.2%)	The essential oil and the major component asaricin cause depressant and ataxia effects in mice.	[151]
*P. solmsianum* C. DC.	Universidade de São Paulo, Brazil	Leaf (HD)	Spathulenol (5.2%), isoelemecin (53.5%)	Antifungal, broth dilution assay (*Cryptococcus neoformans*, MIC 62.5 μg/mL) Antifungal, TLC bioautography (*Cladosporium cladosporioides*, *C. sphareospermum*)	[35,60]
*P. solmsianum* C. DC.	Ubatuba, São Paulo state, Brazil	Leaf (HD)	α-Pinene (22.7%), myrcene (26.1%), δ-3-carene (66.9%), α-selinene (5.5%)	---	[31]
*P. tectoniaefolium* (Kunth) Kunth ex C. DC.	Fazenda Sucupira, Embrapa, Brasília, Brazil	Leaf (HD)	α-Pinene (12.9%), β-pinene (8.8%), caryophyllene oxide (10.9%) ^e^	---	[33]
*P. trigonum* C. DC.	Altos de Campana, Panama	Leaf (HD)	α-Copaene (6.0%), β-elemene (8.4%), β-caryophyllene (7.1%), germacrene D (19.7%), δ-cadinene (7.2%), α-cadinol (5.8%)	---	[47]
*P. tuberculatum* Jacq. var. *tuberculatum*	Rondônia state, Brazil	Leaf (HD)	α-Pinene (8.4%), β-pinene (7.0%), limonene (6.7%), (*E*)-β-ocimene (9.0%), β-caryophyllene (26.3%), (*E*)-β-farnesene (6.1%), α-cadinol (13.7%)	---	[152]
*P. tuberculatum* Jacq.	Universidade Estadual Paulista, Araraquara, São Paulo state, Brazil	Leaf (HD)	α-Pinene (10.4%), β-pinene (12.5%), (*E*)-β-ocimene (8.6%), β-caryophyllene (40.2%), (*E*)-β-farnesene (8.3%), germacrene D (5.5%)	---	[59]
*P. tuberculatum* Jacq.	Universidade Estadual Paulista, Araraquara, São Paulo state, Brazil	Floral (HD) ^a^	α-Pinene (28.7%), β-pinene (38.2%), (*E*)-β-ocimene (9.8%), β-caryophyllene (14.0%)	---	[59]
*P. tuberculatum* Jacq.	Universidade Estadual Paulista, Araraquara, São Paulo state, Brazil	Stem (HD)	α-Pinene (17.3%), β-pinene (27.0%), (*E*)-β-ocimene (14.5%), β-caryophyllene (32.1%)	Antifungal, TLC bioautography (*Cladosporium cladosporioides*, *Cladosporium sphareospermum*)	[59]
*P. tuberculatum* Jacq.	Universidade de São Paulo, Brazil	Leaf (HD)	(*E*)-Nerolidol (12.7%), spathulenol (15.8%), viridiflorol (13.5%), τ-cadinol (6.3%)	---	[35]
*P. umbellatum* L.	Monteverde, Costa Rica	Aerial parts (HD)	β-Elemene (6.9%), β-caryophyllene (28.3%), germacrene D (16.7%), bicyclogermacrene (6.6%), (*E*,*E*)-α-farnesene (14.5%)	---	[49]
*P. umbellatum* L.	Araraquara, São Paulo state, Brazil	Leaf (HD)	γ-Muurolene (8.9%), germacrene D (34.2%), bicyclogermacrene (9.0%), γ-cadinene (5.9%), δ-cadinene (15.0%)	---	[35,60]
*P. umbellatum* L.	Topes de Collantes Nature Reserve, Escambray Mountains, Cuba	Leaf (HD)	Camphor (9.6%), safrole (26.4%), β-caryophyllene (6.6%)	Antioxidant (DPPH radical scavenging assay, IC_50_ 32.3 μg/mL)	[50]
*P. umbellatum* L.	Campinas, São Paulo state, Brazil	Leaf (HD)	β-Caryophyllene (6.3%), germacrene D (55.8%), bicyclogermacrene (11.8%)	---	[31]
*P. variabile* C. DC.	Lachuá, Alta Verapaz, Guatemala	Leaf (HD)	Camphene (16.6%), *p*-cymene (6.3%), limonene (13.9%), camphor (28.4%), guaiol (6.3%)	---	[140]
*P. vicosanum* Yunck.	Parque Estabdual do Rio Doce, Minas Gerais state, Brazil	Aerial parts (HD)	α-Pinene (6.1%), 1,8-cineole (10.4%), limonene (45.5%)	---	[153]
*P. vicosanum* Yunck.	Universidade Federal de Minas Gerais, Belo Horizonte, Minas Gerais state, Brazil	Aerial parts (HD)	α-Pinene (7.2%), 1,8-cineole (15.0%), limonene (40.0%), terpinolene (10.1%)	---	[153]
*P. vicosanum* Yunck.	Dourados, Mato Grosso do Sul, Brazil	Leaf (HD)	Limonene (9.1%), γ-elemene (14.2%), α-alaskene (13.4%)	Anti-inflammatory (rat paw edema, 100–300 mg/kg)	[89]
*P. vitaceum* Yunck.	Manaus-Caracaraí, Amazonas, Brazil	Aerial parts (HD)	*p*-Cymene (12.8%), limonene (33.2%), (*E*)-nerolidol (20.6%), caryophyllene oxide (5.2%)	---	[129]
*P. xylosteoides* (Kunth) Steud.	Fazenda Sucupira, Embrapa, Brasília, Brazil	Leaf (HD)	Myrcene (31.0%), α-terpinene (11.3%), *p*-cymene (12.4%), γ-terpinene (26.1%)	---	[17,33]
*P. xylosteoides* (Kunth) Steud.	São Francisco de Paula, Rio Grande do Sul state, Brazil	Aerial parts (HD)	α-Pinene (6.0%), limonene (5.1%), zingiberene (9.3%), safrole (47.8%)	Acaricidal (*Rhipicephalus* (*Boophilus*) *microplus*, LC_50_ 6.15 μL/mL)	[106]
*P. xylosteoides* (Kunth) Steud.	Orleans, Santa Catarina state, Brazil	Leaf (HD)	α-Pinene (7.7%), safrole (84.1%)	Antibacterial, broth dilution assay (*Bacillus cereus*, MIC 2091 μg/mL; *Staphylococcus aureus*, MIC 2091 μg/mL)	[154]
*P. xylosteoides* (Kunth) Steud.	São Bonifácio, Santa Catarina state, Brazil	Leaf (HD)	α-Pinene (15.3%), safrole (75.8%)	Antibacterial, broth dilution assay (*Bacillus cereus*, MIC 2091 μg/mL; *Staphylococcus aureus*, MIC 2091 μg/mL)	[154]
*P. xylosteoides* (Kunth) Steud.	Ubatuba, São Paulo state, Brazil	Leaf (HD)	Germacrene B (10.6%), *trans*-β-guaiene (7.8%), (*E*)-nerolidol (8.2%), spathulenol (12.3%), β-copaen-4α-ol (9.4%)	---	[31]
*P. xylosteoides* (Kunth) Steud.	Cerro Azul, Paraná state, Brazil	Leaf (HD)	α-Thujene (7.9%), β-phellandrene (22.6%), δ-elemene (6.6%), β-caryophyllene (7.0%), bicyclogermacrene (7.2%), (*E*)-nerolidol (8.5%)	---	[70]

^a^ Floral = Infloresences or infructesence “spikes”. ^b^ The essential oil had two unidentified major components (11.6% and 13.5%). ^c^ Based on retention indices (RI), this analysis is doubtful. γ-Elemene should elute before germacrene D. The compound identified as γ-elemene is probably bicyclogermacrene. γ-Cadinene should elute after germacrene D. The compound identified as γ-cadinene may be γ-muurolene. ^d^ Percentages are based on isolated yields and not by GC integration. ^e^ Only 56.8% of the essential oil composition identified.

## Appendix C

**Table A2:** Chemical classes and concentrations of *Piper* essential oils used for the principal component analysis.

*Piper* Species	Classes (%)						Biological activity	Ref.
	PP	MH	OM	SH	OS	Total		
*P. aducum*	0.0	13.7	40.8	16.8	24.3	95.6	Antiprotozoal	[53]
*P. aduncum*	0.0	20.9	7.5	42.2	18.3	88.9	Antiprotozoal	[70]
*P. aduncum*	87.8	0.9	0.0	6.5	2.4	97.6	Antimicrobial	[30]
*P. aduncum*	0.0	14.1	31.8	26.0	11.6	83.5	Antimicrobial	[35]
*P. aduncum*	0.9	9.7	13.4	19.3	43.9	87.2	Antiprotozoal	[68]
*P. aduncum*	0.0	9.7	50.3	8.3	29.3	97.6	Antiprotozoal	[32]
*P. aduncum*	0.0	13.7	31.7	39.8	11.8	97.0	Antimicrobial	[59]
*P. aduncum*	0.0	55.1	11.8	13.9	10.6	91.4	Antimicrobial	[59]
*P. aduncum*	46.8	25.1	15.7	6.3	1.0	94.8	Antimicrobial	[65]
*P. aduncum*	89.2	1.1	2.2	3.8	2.6	98.9	Insecticidal	[16]
*P. aduncum*	95.2	0.0	0.0	0.0	3.9	99.2	Acaricidal	[28]
*P. aequale*	0.0	29.2	0.0	42.9	20.9	93.0	Citotoxic	[95]
*P. aequale*	3.7	70.2	0.2	12.4	13.5	100.0	Antimicrobial	[34]
*P. angustifolium*	0.0	13.4	4.7	21.9	53.0	93.0	Antiprotozoal	[69]
*P. anonifolium*	0.0	11.7	0.0	38.6	38.9	89.2	Antimicrobial/Enzimaitc	[61]
*P. aleyreanum*	0.0	10.1	0.0	56.7	23.1	89.9	Antimicrobial/Citotoxic	[61]
*P. aleyreanum*	0.0	16.6	16.4	16.2	28.3	77.5	Antinociceptive/Anti-inflammatory	[90]
*P. arboreum*	0.0	0.0	1.7	46.7	41.0	89.4	Antiprotozoal	[70]
*P. auritum*	88.5	3.4	0.7	3.2	0.6	96.4	Antiprotozoal	[117]
*P. biasperatum*	0.0	4.5	0.0	94.9	0.0	99.4	Cytotoxic	[34]
*P. bredemeyeri*	0.0	0.2	0.0	99.8	0.0	100.0	Antimicrobial	[34]
*P. caldense*	0.0	0.0	47.1	7.2	24.3	78.6	Antimicrobial	[54]
*P. caldense*	0.0	0.0	6.5	17.2	59.7	83.4	Antimicrobial	[54]
*P. caldense*	0.0	0.4	0.0	63.5	20.1	84.0	Antimicrobial	[54]
*P. callosun*	80.1	6.9	4.2	4.9	2.1	98.2	Enzyme inhibitory	[62]
*P. cernuum*	0.0	18.9	0.0	62.4	16.7	97.9	Antimicrobial	[38]
*P. cernuum*	0.0	3.1	0.0	81.3	15.0	99.4	Antimicrobial	[60]
*P. cernuum*	0.0	0.0	0.0	78.9	0.0	78.9	Cytotoxic	[39]
*P. cernuum*	0.0	52.2	23.2	0.0	0.0	75.4	Cytotoxic	[96]
*P. cernuum*	0.0	20.5	0.0	31.0	35.0	86.5	Antiprotozoal/Antimicrobial	[70]
*P. cernuum*	0.0	12.3	1.2	10.4	75.5	99.4	Antimicrobial	[37]
*P. claussenianum*	0.0	0.3	0.0	9.1	86.2	95.6	Antiprotozoal/Antimicrobial	[71,126]
*P. claussenianum*	0.0	1.5	51.4	7.5	28.3	88.7	Antiprotozoal/Antimicrobial	[71,126]
*P. corcovadensis*	30.6	35.1	0.2	20.4	6.4	92.7	Insecticidal	[127]
*P. crassinervium*	0.0	7.8	0.0	54.8	37.1	99.7	Antimicrobial	[60]
*P. demeraranum*	0.0	29.9	0.0	63.0	0.0	92.9	Antiprotozoal	[72]
*P. diospyrifolium*	0.0	19.5	1.1	68.2	11.2	100.0	Antimicrobial	[55]
*P. diospyrifolium*	0.0	16.1	0.0	46.5	28.5	91.1	Antiprotozoal	[70]
*P. divaricatum*	89.6	3.3	0.0	5.6	0.6	99.1	Antimicrobial	[40]
*P. divaricatum*	98.0	0.0	0.0	0.0	0.0	98.0	Antimicrobial	[44]
*P. divaricatum*	89.1	7.2	0.1	1.9	0.2	98.5	Antimicrobial	[42]
*P. duckei*	0.0	1.1	5.8	60.2	23.0	90.1	Antiprotozoal	[72]
*P. fimbriulatum*	0.0	19.5	0.0	76.4	4.1	100.0	Antimicrobial	[34]
*P. gaudichaudianum*	0.0	2.4	0.0	44.3	44.0	90.8	Insecticidal	[136]
*P. gaudichaudianum*	0.0	0.1	0.1	65.4	28.3	93.8	Cytotoxic	[138]
*P. gaudichaudianum*	0.1	4.2	0.4	56.0	29.5	90.2	Cytotoxic	[137]
*P. gaudichaudianum*	0.0	0.9	0.0	72.6	14.4	87.9	Antimicrobial	[35]
*P. gaudichaudianum*	0.0	7.1	0.0	76.0	9.9	93.0	Antiprotozoal	[70]
*P. glabratum*	0.2	25.8	1.0	50.4	21.2	98.6	Anti-inflammatory	[88]
*P. glabrescens*	0.0	83.7	0.0	15.3	1.0	100.0	Cytotoxic	[34]
*P. hispidinervum*	85.5	9.3	0.0	2.5	0.8	98.0	Antimicrobial	[139]
*P. hispidum*	0.0	18.5	1.0	52.2	16.6	88.3	Antimicrobial/Enzyme inhibitory	[61]
*P. hispidum*	0.0	43.9	1.7	27.8	15.4	88.8	Antimicrobial/Cytotoxic	[97]
*P. hispidum*	58.3	5.0	12.6	14.2	5.0	95.1	Insecticidal	[47]
*P. hostmannianum*	57.0	1.0	5.6	20.1	10.6	94.3	Insecticidal	[136]
*P. humaytanum*	0.0	1.5	0.0	34.0	45.4	80.9	Insecticidal	[136]
*P. ilheuense*	0.0	0.0	0.0	46.5	34.1	80.6	Antimicrobial	[57]
*P. imperiale*	6.7	2.7	0.0	89.4	1.2	100.0	Antimicrobial/Cytotoxic	[34]
*P. klotzschianum*	98.5	0.0	0.0	0.6	0.5	99.6	Insecticidal	[141]
*P. klotzschianum*	39.4	44.3	0.0	14.5	0.0	98.2	Insecticidal	[141]
*P. longispicum*	1.1	1.4	0.2	66.0	11.9	80.6	Insecticidal	[47]
*P. marginatum*	0.0	1.4	0.7	2.2	26.2	30.5	Insecticidal	[146]
*P. marginatum*	28.4	0.0	0.0	44.6	26.2	99.2	Insecticidal	[146]
*P. marginatum*	51.6	0.0	0.0	21.7	26.4	99.7	Insecticidal	[146]
*P. marginatum*	42.0	10.3	1.6	17.6	17.5	89.0	Antimicrobial/Enzyme inhibitory	[62]
*P. mikanianum*	67.9	0.5	0.0	23.4	8.6	100.4	Acaricidal	[106]
*P. mollicomum*	0.6	24.2	9.8	33.2	25.1	92.9	Antinociceptive	[91]
*P. mosenii*	0.0	7.2	0.0	41.5	37.7	86.4	Antiprotozoal/Antimicrobial	[70]
*P. oblanceolatum*	0.0	16.6	12.2	61.4	9.8	100.0	Antimicrobial/Cytotoxic	[34]
*P. permucronatum*	98.8	0.8	0.0	0.0	0.0	99.6	Insecticidal	[136]
*P. regnellii*	0.0	60.8	17.8	13.8	6.1	98.5	Antimicrobial	[38]
*P. regnellii*	0.3	0.0	0.4	82.0	10.8	93.5	Cytotoxic	[98]
*P. rivinoides*	0.0	65.9	0.8	21.8	4.8	93.2	Antinociceptive	[91]
*P. rivinoides*	0.0	10.4	0.0	54.7	20.1	85.2	Antiprotozoal	[70]
*P. solmsianum*	40.3	30.3	0.0	5.8	4.2	80.7	Depressant/Ataxia	[151]
*P. solmsianum*	53.5	0.0	0.0	12.4	12.3	78.2	Antimicrobial	[35]
*P. tuberculatum*	0.0	35.7	0.3	60.2	2.9	99.1	Antimicrobial	[59]
*P. vicosanum*	0.0	16.4	0.0	62.6	20.8	99.8	Anti-inflammatory	[89]
*P. xylosteoides*	48.5	17.0	0.4	23.7	10.4	100.0	Acaricidal	[106]
